# Inverse Hypercorroles

**DOI:** 10.1021/acs.inorgchem.4c00344

**Published:** 2024-05-02

**Authors:** W. Ryan Osterloh, Nicolas Desbois, Jeanet Conradie, Claude P. Gros, Karl M. Kadish, Abhik Ghosh

**Affiliations:** † ICMUB (UMR CNRS 6302), Université de Bourgogne, 9, Avenue A. Savary, BP 47870, 21078 Dijon Cedex, France; ‡ Department of Chemistry, UiT − The Arctic University of Norway, N-9037 Tromso̷, Norway; § Department of Chemistry, University of the Free State, 9300 Bloemfontein, Republic of South Africa; ∥ Department of Chemistry, University of Houston, Houston, Texas 77204-5003, United States

## Abstract

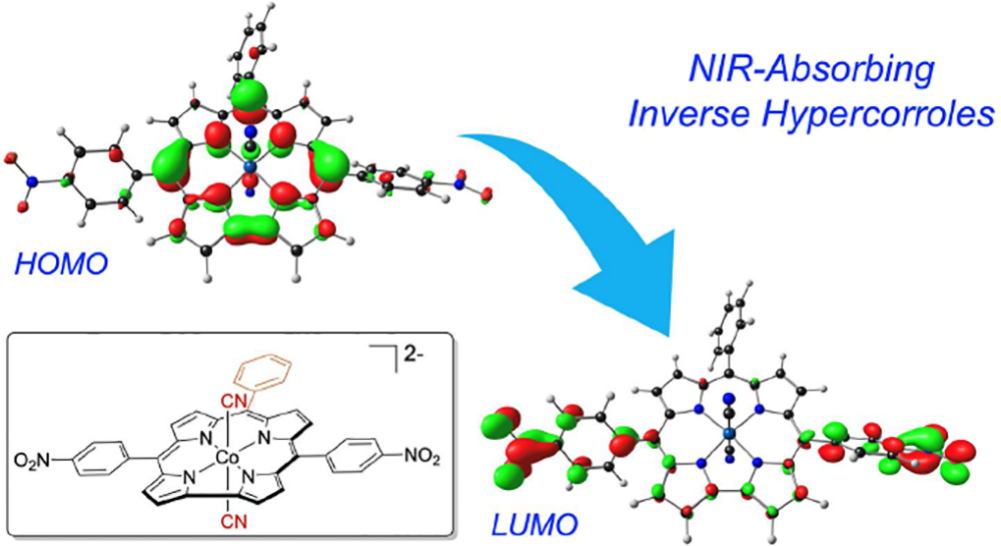

Ground-state and time-dependent density functional theory (TDDFT) calculations with the long-range-corrected, Coulomb-attenuating CAMY-B3LYP exchange-correlation functional and large, all-electron STO-TZ2P basis sets have been used to examine the potential “inverse hypercorrole” character of *meso-p*-nitrophenyl-appended dicyanidocobalt(III) corrole dianions. The effect is most dramatic for 5,15-bis(*p*-nitrophenyl) derivatives, where it manifests itself in intense NIR absorptions. The 10-aryl groups in these complexes play a modulatory role, as evinced by experimental UV–visible spectroscopic and electrochemical data for a series of 5,15-bis(*p*-nitrophenyl) dicyanidocobalt(III) corroles. TDDFT (CAMY-B3LYP) calculations ascribe these features clearly to a transition from the corrole’s a_2u_-like HOMO (retaining the *D*_4h_ irrep used for metalloporphyrins) to a nitrophenyl-based LUMO. The outward nature of this transition contrasts with the usual phenyl-to-macrocycle direction of charge transfer transitions in many hyperporphyrins and hypercorroles; thus, the complexes studied are aptly described as inverse hypercorroles.

## Abstract 2

TDDFT calculations lend support to the idea of “inverse hypercorroles”, in which outward corrole-to-*meso*-aryl transitions result in intense NIR absorption bands.

## Introduction

Gouterman’s four-orbital model conceptualizes the classic optical spectra of porphyrins as transitions from two near-degenerate highest occupied molecular orbitals (HOMOs), which transform as a_1u_ and a_2u_ in a *D*_4h_ metalloporphyrin, to two degenerate lowest unoccupied molecular orbitals (LUMOs), which transform as e_g_.^1−5^ In a “normal” porphyrin, these four molecular orbitals (MOs) are well-separated from all other occupied and unoccupied orbitals. Hyperporphyrins are a diverse class of porphyrin derivatives with red-shifted optical spectra in which the frontier orbitals are modified in one of a myriad ways that lower the HOMO–LUMO gap.^6^ Two common mechanisms underlying hyper spectra involve admixture of transition metal d orbitals or of substituent-based orbitals into porphyrin-based MOs. In certain cases, the very identity of the HOMOs and LUMOs may be altered, and these may correspond to orbitals unrelated to the four-orbital model. A classic example of the latter scenario is found in diprotonated *meso*-tetrakis(*p*-aminophenyl)porphyrin, [H_4_TAPP]^2+^ in which the HOMO is almost exclusively localized on the electron-rich *p*-aminophenyl groups.^7−10^ The lowest-energy transition in this system is then an aminophenyl-to-porphyrin charge transfer transition. As it happens, charge transfer transitions underlie many, if not most, cases of hyperporphyrin spectra.^6^

In the early days of corrole chemistry,^11−14^ it was shown that simple corrole derivatives also conform to the four-orbital model.^15^ Soon, several cases of hypercorroles emerged, consisting of noninnocent transition metal *meso*-triarylcorroles in which the major optical transitions are thought to involve a significant degree of aryl-to-corrole charge transfer character.^16,17^ Protonated *meso*-tris(*p*-aminophenyl)corrole, an analogue of [H_4_TAPP]^2+^, was also shown to exhibit a hyper spectrum.^18^ In a recent Perspective article,^6^ Wamser and Ghosh considered the possibility of what might be termed *inverse hyper spectra* in which *outward* electron flow from a porphyrin or corrole core to the *meso*-aryl substituents results in strongly red-shifted optical spectra. Recently, Osterloh et al.^19^ have suggested, based on UV–vis-NIR absorption and electrochemical evidence, that *meso*-nitrophenyl-appended dicyanidocobalt(III) corrole dianions should qualify as inverse hypercorroles. In the absence of modern quantum chemical studies, however, the theoretical basis of the inverse hypercorrole description has remained uncertain and speculative.

**Scheme 1:**
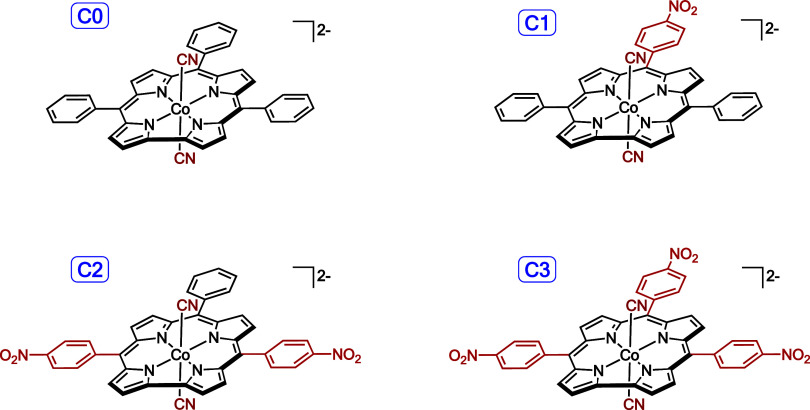
Structures of Species Computationally Modeled in This Study In the **Cn** notation used, **C** refers ‘*c*omputational modeling’ and the numeral **n** to the number of nitrophenyl groups in the species.

We accordingly undertook a state-of-the-art ground-state and time-dependent density functional theory (DFT and TDDFT^20,21^) study of four *meso*-nitrophenyl-appended dicyanidocobalt(III) corroles (Scheme 1), namely {Co[TPC](CN)_2_}^2–^ (**C0**), {Co[(5,15-P)(10-*p*NO_2_P)C](CN)_2_}^2–^ (**C1**), {Co[(5,15-*p*NO_2_P)(10-P)C](CN)_2_}^2–^ (**C2**), and {Co[T*p*NO_2_PC](CN)_2_}^2–^ (**C3**), where P = *p*henyl, C = *c*orrole, and TPC = *t*ri*p*henyl*c*orrole. As described below, the results confirm the formulation of these systems as authentic inverse hypercorroles. Additional experimental data on the effects of substituents on the hypercorrole spectra are also included.

## Experimental Section

### Starting Materials

All chemicals and solvents were of the highest grade available and were used without further purification. Benzonitrile (PhCN) was purchased from Sigma-Aldrich and distilled from P_4_O_10_ under a vacuum prior to use. Tetra-*n*-butyl-ammonium perchlorate (TBAP, for electrochemical analysis, ≥ 99.0%) and 95.0% tetra-*n*-butyl-ammonium cyanide (TBACN, 95%) were purchased from Sigma-Aldrich and stored in a desiccator until used.

### UV–Visible Spectroscopy

UV–visible spectra of the synthesized compounds were recorded on a Varian Cary 50 or a Hewlett-Packard model 8453 diode array spectrophotometer, and quartz cells with an optical path length of 10 mm were used.

### NMR Spectroscopy

^1^H NMR spectra were recorded in CDCl_3_ on a Bruker AVANCE NEO spectrometer (400 and 500 MHz). The measurements were made at the PACSMUB-WPCM technological platform, which relies on the “*Institut de Chimie Moléculaire de l’Université de Bourgogne*” and Welience “TM”, a Burgundy University private subsidiary. For DMSO-ligated cobalt corroles, gaseous NH_3_ was added to enhance the resolution of the spectra.

### Mass Spectrometry

Mass spectra were recorded on a Bruker Microflex LRF MALDI Tandem TOF Mass Spectrometer using dithranol as the matrix or on an LTQ Orbitrap XL (Thermo) instrument in the ESI mode (for the HRMS spectra). Corroles **S1** and **S4** were prepared as described in the literature.^19^

### General Procedure for the Synthesis of Free-Base Cobalt Corroles

The 5-(4-nitrophenyl)dipyrromethane (5.62 mmol, 1 equiv) and the appropriate benzaldehyde (2.81 mmol, 0.5 equiv) were dissolved in 560 mL of methanol. Then, a solution of HCl (36%, 28.0 mL) in H_2_O (560 mL) was added, and the reaction mixture was stirred at room temperature for 2 h. The mixture was extracted with chloroform, and the organic phase was washed three times with water, dried, and completed to 1.5L. *p-*Chloranil (1.5 equiv) was added, and the reaction mixture was stirred overnight at room temperature protected from light. Then 7.0 mL of hydrazine was added, and the mixture was further stirred for 30 min. After that, the solvent was evaporated and filtered on a dicalite plug. The compound thus obtained was purified by silica column with CHCl_3_ as the eluent. The crude compound was further recrystallized with DCM and heptane to afford crystal powder. The solid was filtered and dried under vacuum.

### General Procedure for the Synthesis of Mono-DMSO Cobalt Corroles

**Scheme 2:**
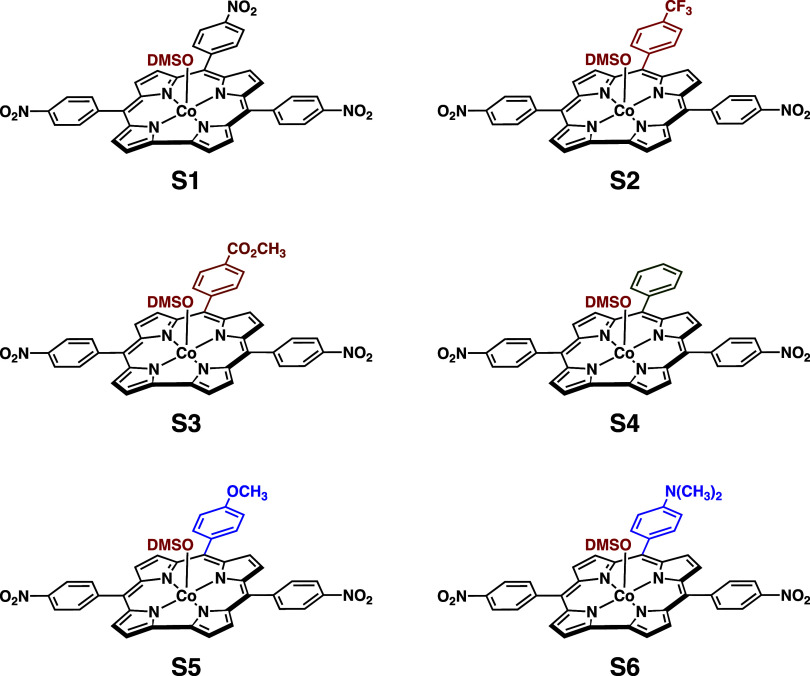
Mono-DMSO Cobalt Corroles Employed in This Study

Each free-base corrole (1.0 equiv) was added to a solution of cobalt acetate tetrahydrate (1.2 equiv) in DMSO (20 mL) in a round-bottom flask, after which the reaction mixture was stirred at 80 °C for 40 min and then cooled to room temperature. The crude mixture was poured into a cold NaCl aqueous solution (0.8 M), the resulting suspension was filtered, and the desired mono-DMSO cobalt corrole (Scheme 2) was washed five times with water (centrifugation) and dried overnight under vacuum.

### Metallocorrole **S2**

The free-base corrole was synthesized according to a published procedure.^22^ The mono-DMSO metallocorrole **S2** was synthesized according to the general procedure starting from 100.1 mg of free-base corrole in 96% yield (115.1 mg). UV–visible (DCM): λ_max_ (ε x 10^–3^ M^–1^ cm^–1^) 386.9 (70.24), 561.0 (13.47) nm. ^1^H NMR (500 MHz, CDCl_3_ + NH_3_ (g)) δ (ppm) 9.26 (d, ^3^*J*_H–H_ = 4.5 Hz, 2H), 8.99 (d, ^3^*J*_H–H_ = 4.5 Hz, 2H), 8.81 (m, 4H), 8.63 (d, ^3^*J*_H–H_ = 8.5 Hz, 4H), 8.46 (d, ^3^*J*_H–H_ = 8.5 Hz, 4H), 8.32 (d, ^3^*J*_H–H_ = 8.0 Hz, 2H), 7.99 (d, ^3^*J*_H–H_ = 8.0 Hz, 2H), 2.57 (s, 6H), −6.59 (s, 6H). ^19^F NMR (470 MHz, CDCl_3_ + NH_3_ (g)) δ −61.89 (s, 3F). MS (MALDI-TOF) *m*/*z* = 740.05 [M-DMSO]^+.^, 740.08 calcd for C_38_H_20_CoF_3_N_6_O_4_. HR-MS (ESI): *m*/*z* = 740.0822 [M-DMSO]^+.^, 740.0825 calcd for C_38_H_20_CoF_3_N_6_O_4_.

### Metallocorrole **S3**

The free-base corrole was synthesized according to a published procedure.^23^ The mono-DMSO metallocorrole **S3** was synthesized according to the general procedure starting from 50 mg of free-base corrole in 92% yield (55.3 mg). UV–visible (DCM): λ_max_ (ε x 10^–3^ M^–1^ cm^–1^) 390.0 (55.24), 560.0 (10.16) nm. ^1^H NMR (500 MHz, CDCl_3_ + NH_3_ (g)) δ (ppm) 9.30 (d, ^3^*J*_H–H_ = 4.5 Hz, 2H), 9.02 (d, ^3^*J*_H–H_ = 4.5 Hz, 2H), 8.89 (d, ^3^*J*_H–H_ = 4.5 Hz, 2H), 8.85 (d, ^*3*^*J*_H–H_ = 4.5 Hz, 2H), 8.66 (d, ^3^*J*_H–H_ = 8.5 Hz, 4H), 8.49 (d, ^3^*J*_H–H_ = 8.5 Hz, 4H), 8.44 (d, ^3^*J*_H–H_ = 8.0 Hz, 2H), 8.32 (d, ^3^*J*_H–H_ = 8.0 Hz, 2H), 4.09 (s, 3H), 2.60 (s, 6H), −6.72 (s, 6H). MS (MALDI-TOF) *m*/*z* = 730.21 [M-DMSO]^+.^, 730.10 calcd for C_39_H_23_CoN_6_O_4_. HR-MS (ESI): *m*/*z* = 730.1035 [M-DMSO]^+.^, 730.1006 calcd for C_39_H_23_CoN_6_O_4_.

### Metallocorrole **S5**

The free-base corrole was synthesized according to the general procedure using 341.3 μL of 4-methoxybenzaldehyde. Yield: 42% (76.3 mg). UV–visible (DCM): λ_max_ (ε × 10^–3^ M^–1^cm^–1^) 442.0 (52.54), 594.0 (16.72), 656.0 (17.13) nm. ^1^H NMR (500 MHz, CDCl_3_ + NH_3_ (g)) δ (ppm) 9.07 (d, ^3^*J*_H–H_ = 4.5 Hz, 2H), 8.86 (d, ^3^*J*_H–H_ = 4.5 Hz, 2H), 8.69 (d, ^3^*J*_H–H_ = 8.5 Hz, 4H), 8.66 (d, ^3^*J*_H–H_ = 4.5 Hz, 2H), 8.62 (d, ^3^*J*_H–H_ = 4.5 Hz, 2H), 8.55 (d, ^3^*J*_H–H_ = 8.5 Hz, 4H), 8.09 (d, ^3^*J*_H–H_ = 8.0 Hz, 2H), 7.32 (d, ^3^*J*_H–H_ = 8.0 Hz, 2H), 4.10 (s, 3H). MS (MALDI-TOF) *m*/*z* = 646.20 [M]^+.^, 646.20 calcd for C_38_H_26_N_6_O_5_. HR-MS (ESI): *m*/*z* = 647.2032 [M + H]^+^, 647.2037 calcd for C_38_H_27_N_6_O_5_.

The mono-DMSO metallocorrole **S5** was synthesized according to the general procedure from 50.3 mg of free-base corrole in 91% yield (55.4 mg). UV–visible (DCM): λ_max_ (ε × 10^–3^ M^–1^cm^–1^) 391.0 (47.38), 563.0 (4.43) nm. ^1^H NMR (400 MHz, CDCl_3_ + NH_3_ (g)) δ (ppm) 9.31 (d, ^3^*J*_H–H_ = 4.5 Hz, 2H), 9.02 (d, ^3^*J*_H–H_ = 4.5 Hz, 2H), 8.95 (d, ^3^*J*_H–H_ = 4.5 Hz, 2H), 8.85 (d, ^3^*J*_H–H_ = 4.5 Hz, 2H), 8.66 (d, ^3^*J*_H–H_ = 8.5 Hz, 4H), 8.51 (d, ^3^*J*_H–H_ = 8.5 Hz, 4H), 8.14 (d, ^3^*J*_H–H_ = 8.0 Hz, 2H), 7.31 (d, ^3^*J*_H–H_ = 8.0 Hz, 2H), 4.10 (s, 3H), 2.61 (s, 6H), −6.82 (s, 6H). MS (MALDI-TOF) *m*/*z* = 702.13 [M-DMSO]^+.^, 702.11 calcd for C_38_H_23_CoN_6_O_5_. HR-MS (ESI): *m*/*z* = 702.1053 [M-DMSO]^+.^, 702.1056 calcd for C_38_H_23_CoN_6_O_5_.

### Metallocorrole **S6**

The free-base corrole was synthesized according to the general procedure using 418.7 mg of 4-(dimethylamino)benzaldehyde. Yield: 3.3% (61.9 mg). UV–visible (DCM): λ_max_ (ε × 10^–3^ M^–1^ cm^–1^) 446.9 (45.06), 598.0 (14.02), 666.9 (15.9) nm. ^1^H NMR (500 MHz, CDCl_3_ + NH_3_ (g)) δ (ppm) 9.01 (d, ^3^*J*_H–H_ = 4.5 Hz, 2H), 8.81 (d, ^3^*J*_H–H_ = 4.5 Hz, 2H), 8.69 (d, ^3^*J*_H–H_ = 4.5 Hz, 2H), 8.66 (d, ^3^*J*_H–H_ = 8.5 Hz, 4H), 8.58 (d, ^3^*J*_H–H_ = 4.5 Hz, 2H), 8.54 (d, ^3^*J*_H–H_ = 8.5 Hz, 4H), 8.04 (d, ^3^*J*_H–H_ = 8.0 Hz, 2H), 7.11 (d, ^3^*J*_H–H_ = 8.0 Hz, 2H), 3.22 (s, 6H). MS (MALDI-TOF) *m*/*z* = 659.13 [M]^+.^, 659.23 calcd for C_39_H_29_N_7_O_4_. HR-MS (ESI): *m*/*z* = 660.2352 [M + H]^+^, 660.2354 calcd for C_39_H_30_N_7_O_4_.

The mono-DMSO metallocorrole **S6** was synthesized according to the general procedure starting from 19.5 mg of free-base corrole in 42% yield (9.8 mg). UV–visible (DCM): λ_max_ (ε × 10^–3^ M^–1^cm^–1^) 390.0 (25.02), 568.1 (5.22), nm. ^1^H NMR (500 MHz, CDCl_3_ + NH_3_ (g)) δ (ppm) 9.27 (d, ^3^*J*_H–H_ = 4.5 Hz, 2H), 8.99 (m, 4H), 8.83 (d, ^3^*J*_H–H_ = 4.5 Hz, 2H), 8.64 (d, ^3^*J*_H–H_ = 8.5 Hz, 4H), 8.49 (d, ^3^*J*_H–H_ = 8.5 Hz, 4H), 8.08 (d, ^3^*J*_H–H_ = 8.0 Hz, 2H), 7.13 (d, ^3^*J*_H–H_ = 8.0 Hz, 2H), 3.22 (s, 6H), 2.59 (s, 6H), −6.70 (s, 6H). MS (MALDI-TOF) *m*/*z* = 715.18 [M-DMSO]^+.^, 715.14 calcd for C_39_H_26_CoN_7_O_4_. HR-MS (ESI): *m*/*z* = 715.1376 [M-DMSO]^+.^, 715.1373 calcd for C_39_H_26_CoN_7_O_4_.

### General Procedure for the Generation of Dicyanidocobalt Corroles

Dicyanidocobalt corrole dianions were generated in situ by dissolving the mono-DMSO metallocorroles **S1**–**S6** in benzonitrile containing 0.1 M tetra(*n*-butyl)ammonium perchlorate (TBAP) followed by the addition of 100 equiv of tetra(*n*-butyl)ammonium cyanide (TBACN).

### Electrochemistry

Cyclic voltammetry was carried out at 298 K in benzonitrile (purified as described as earlier^24^) using an EG&G Princeton Applied Research (PAR) 173 potentiostat/galvanostat. A homemade three-electrode cell was used for all electrochemical measurements. A three-electrode system was used in each case and consisted of a glassy carbon working electrode. A platinum wire served as the auxiliary electrode and a saturated calomel electrode as the reference electrode, which was separated from the bulk of the solution by means of a salt bridge of low porosity which contained the solvent-supporting electrolyte (TBAP) mixture.

### DFT and TDDFT Calculations

Geometry optimizations were carried out with scalar-relativistic DFT using the zeroth order regular approximation (ZORA^25^) to the Dirac equation, the OLYP^26,27^ functional augmented with the Grimme’s D3^28,29^ dispersion correction, all-electron Slater-type ZORA TZ2P basis sets, fine integration grids, and tight criteria for the SCF cycles and geometry optimizations, as implemented in the ADF program system.^30^ A *C*_2_ symmetry constraint was used for all four species studied (Scheme 2). Solvation was modeled with COSMO (conductor-like screening model^31−34^) with acetonitrile as the solvent. The optimized geometries so obtained were used for range-separated TDDFT calculations with the CAMY-B3LYP^35−37^ exchange-correlation functionals and the same basis sets.

## Results

### UV–Vis–NIR and Electrochemical Studies

One of the most notable cases of hypercorrole spectra has been recently documented for dicyanidocobalt(III) 5,15-bis(*p*-nitrophenyl)corroles and 5,10,15-tris(*p*-nitrophenyl)corrole.^19^ The complexes exhibit an intense absorption in the 780–850 nm range, which is absent in analogous bis-cyano ligated triarylcorrole complexes that do not carry *p*-nitrophenyl groups at the lateral 5,15-*meso*-substituents. Thus, a 10-(*p*-nitrophenyl) substituent, by itself, does not lead to a similarly striking hypercorrole spectrum.^38−40^ In the same vein, 5,15-(*m*-nitrophenyl) substituents do not quite lead to a dramatic hypercorrole spectrum.^19^ Herein, we have further clarified the role of 10-substituents via the examination of a series of 5,15-bis(*p*-nitrophenyl)-10-(*p*-X-phenyl)corrole complexes, {Co^III^[(*p*NO_2_P)_2_(*p*XP)C](CN)}^2–^, where the *para* substituent (X) at the 10-position ranges across those shown in Scheme 1.

**Figure 1:**
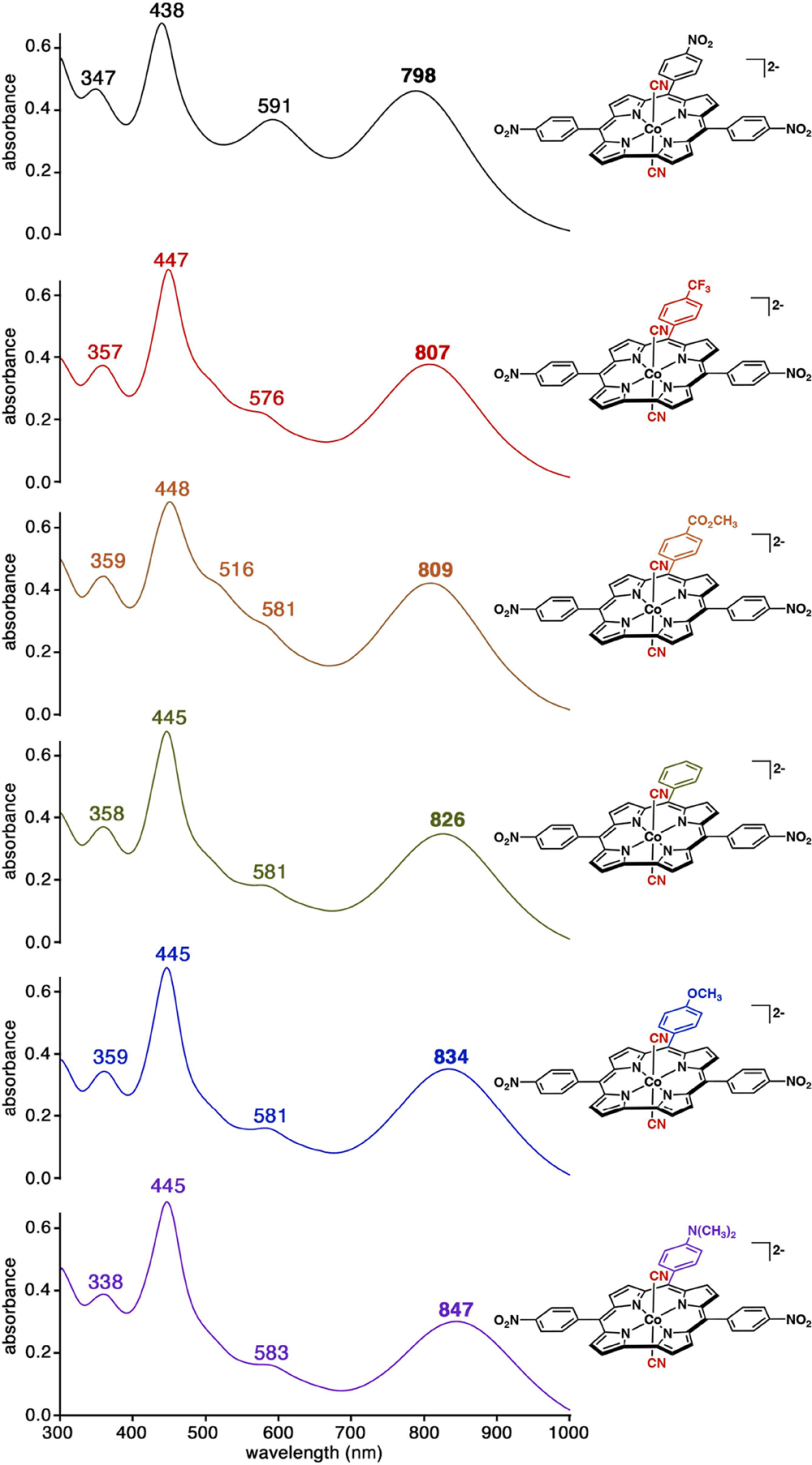
UV–vis spectra of cobalt 5,15-di(4-nitrophenyl)corroles (at ∼10^–5^ M) all in PhCN containing 0.1 M TBAP with 100 equiv of added TBACN.

**Figure 2:**
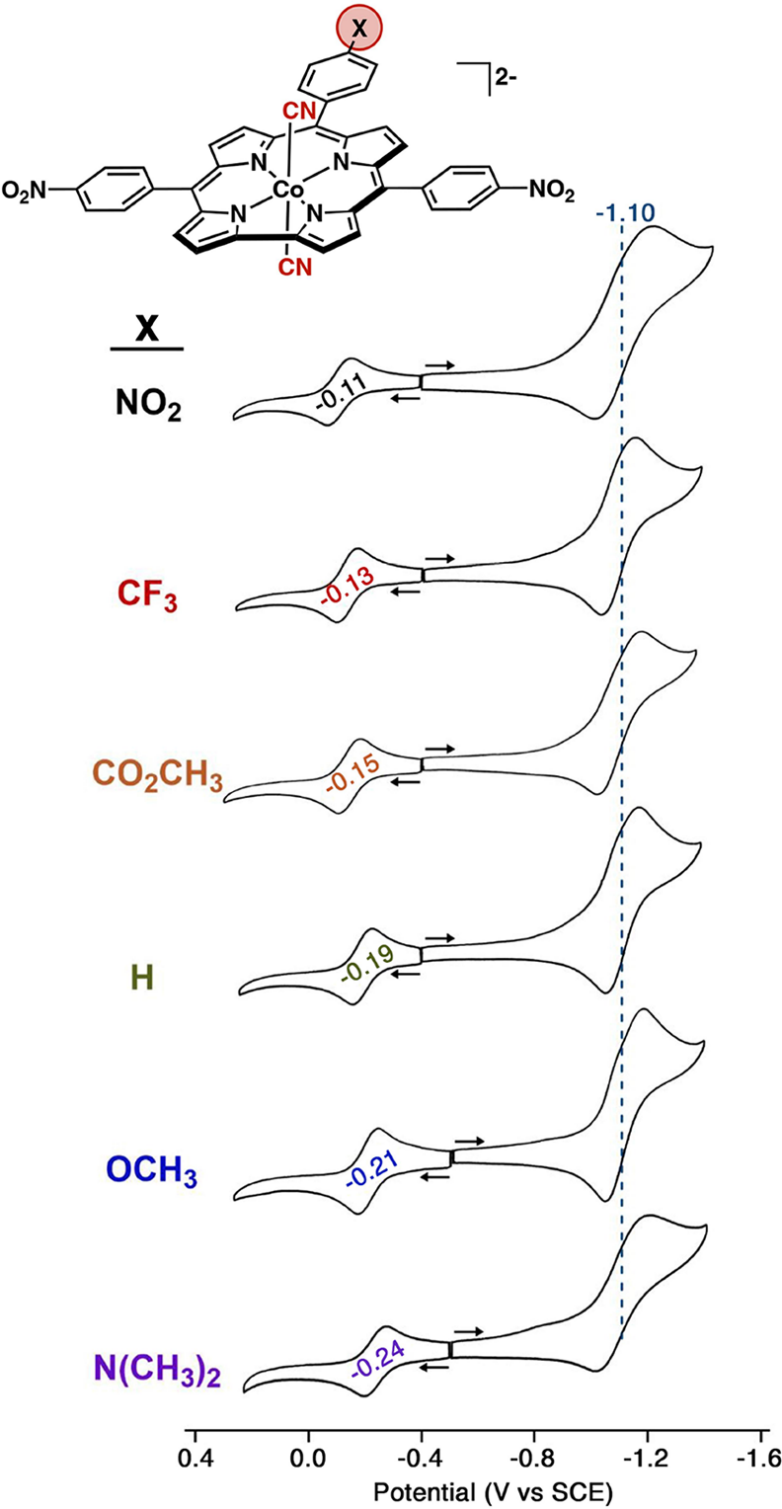
Cyclic voltammograms of cobalt 5,15-di(4-nitrophenyl)corroles in PhCN/0.1 M TBAP with 100 equiv of added TBACN. The reduction process at −1.10 V in blue corresponds to overlapping electron additions at the two or three *meso*-nitrophenyl groups. Scan rate: 0.1 V/s.

**Figure 3:**
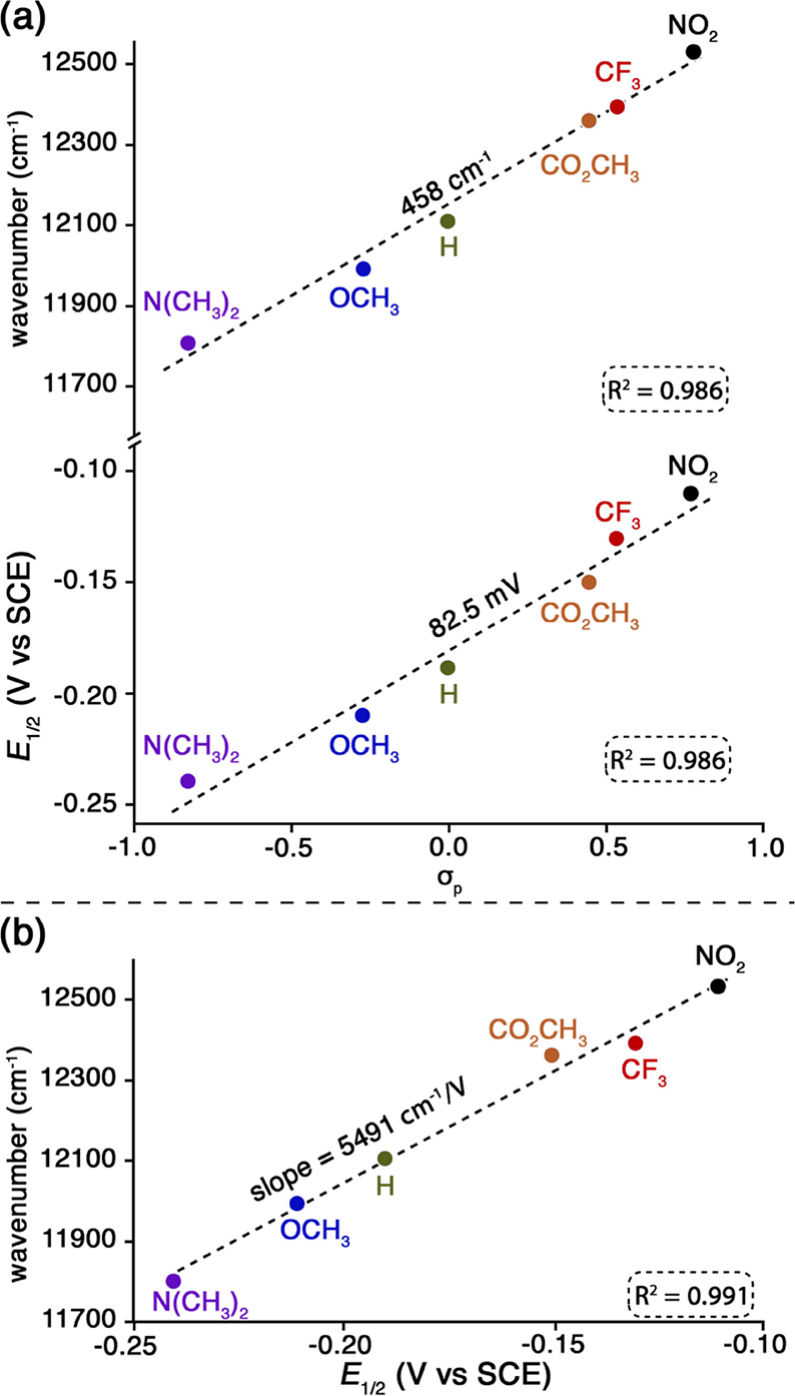
(a) Hammett plots for the lowest-energy absorption band (above) and *E*_1/2_ for the first oxidation process (below), for measurements in 0.1 M TBAP in PhCN with 100 equiv of added TBACN and (b) plot of wavenumber for the lowest energy absorption band vs *E*_1/2_ for the first oxidation process in PhCN/0.1 M TBAP with 100 equiv of added TBACN.

As shown in Figure 1 and summarized in Table 1, the lowest-energy absorption for the dicyanidocobalt(III) complexes in benzonitrile was found to shift from 798 nm for X = NO_2_ to 847 nm for X = NMe_2_ (Table 1 and Figure 1). Cyclic voltammetry measurements suggest that the shift reflects a modest elevation in the orbital energy of the HOMO by about 130 mV, going from X = NO_2_ to X = NMe_2_, while the LUMO, presumably localized in the 5,15-*p*-nitrophenyl groups, remains essentially constant in terms of orbital energy (Figure 2). As discussed below, DFT calculations nicely confirm this conclusion. Furthermore, both the energy of the lowest-energy NIR absorption band and the oxidation potential increase linearly with the Hammett substituent constants of the *para* substituents, with excellent correlation coefficients (Figure 3a). The linear shifts of the oxidation potentials with σ_para_ strongly suggest that HOMO has the same qualitative character across all of the species studied. Understandably, the NIR absorption energies and oxidation potentials also exhibit a linear relationship (Figure 3b).

**Table 1:** UV–visible Spectral Data for Dicyanidocobalt(III) 5,15-Bis(*p*-nitrophenyl)-10-(*p*-X-phenyl)corroles Generated In Situ from Precursors **S1**–**S6** (See Scheme 2 for Structures) and TBACN (100 equiv) in PhCN^a^

			λ, nm (ε × 10^–4^ M^–1^ cm^–1^)		
precursor	X^b^	σ_p_	visible region		NIR region
**S1**	NO_2_	0.78	438 (3.1)	591 (1.8)	**798** (2.2)
**S2**	CF_3_	0.54	447 (3.3)	576 (1.2)	**807** (1.9)
**S3**	CO_2_CH_3_	0.45	448 (3.2)	581 (1.2)	**809** (2.0)
**S4**	H	0.00	445 (3.3)	581 (1.0)	**826** (1.8)
**S5**	OCH_3_	–0.27	445 (3.4)	581 (0.9)	**834** (1.8)
**S6**	N(CH_3_)_2_	–0.83	445 (3.4)	583 (0.9)	**847** (1.6)

a The diagnostic inverse hypercorrole maxima are indicated in bold.

b X = the *para* substituent at the 10-*meso* phenyl position.

### DFT and TDDFT Calculations

**Figure 4:**
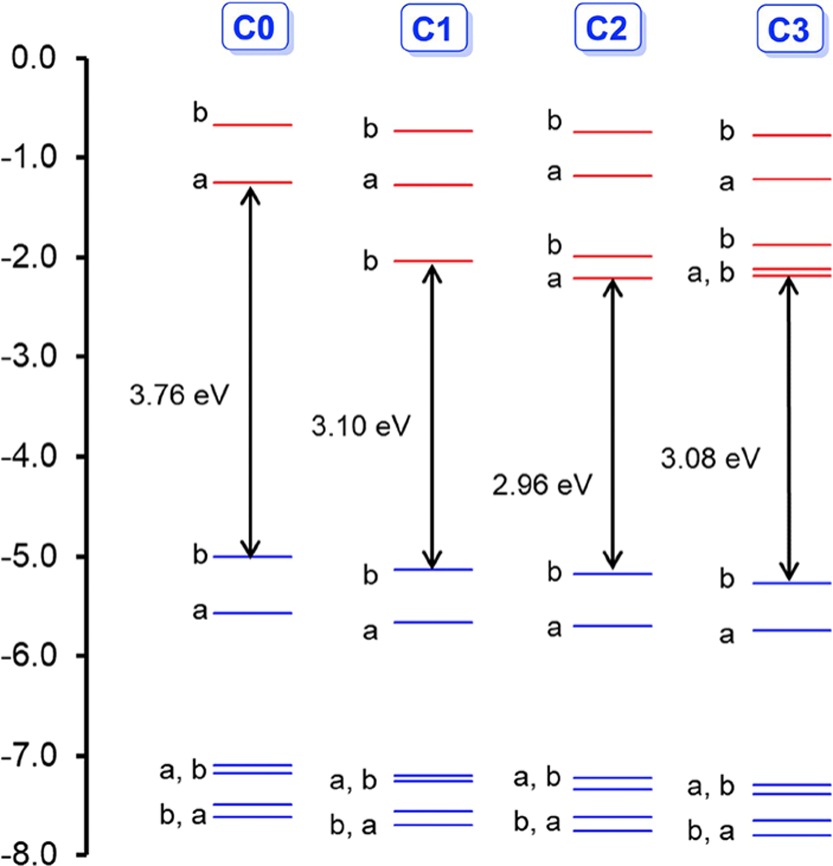
CAMY-B3LYP-D3/STO-TZ2P-COSMO frontier MO energy levels along with C_2_ irreps (a and b).

**Figure 5:**
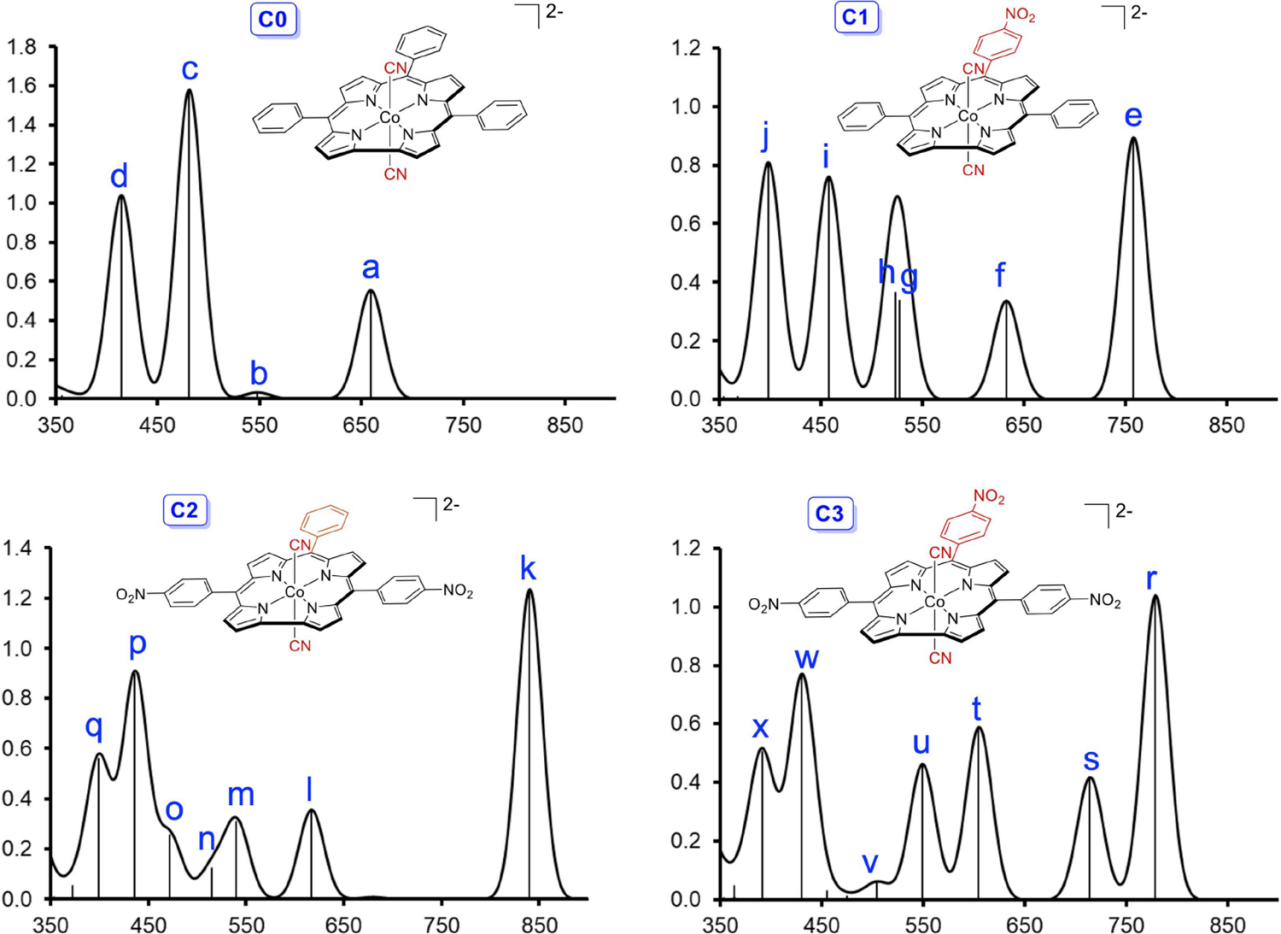
Simulated TD-CAMY-B3LYP-D3/STO-TZ2P-COSMO optical spectra (oscillator strengths vs wavelength in nm) in dichloromethane. The vertical lines represent calculated transitions which have then been broadened with Gaussians to generate the simulated spectra. The peak labels are cross-referenced in Table 2, which lists the MO compositions of the peaks in question. The MOs themselves are visually depicted in Figure 6.

**Figure 6:**
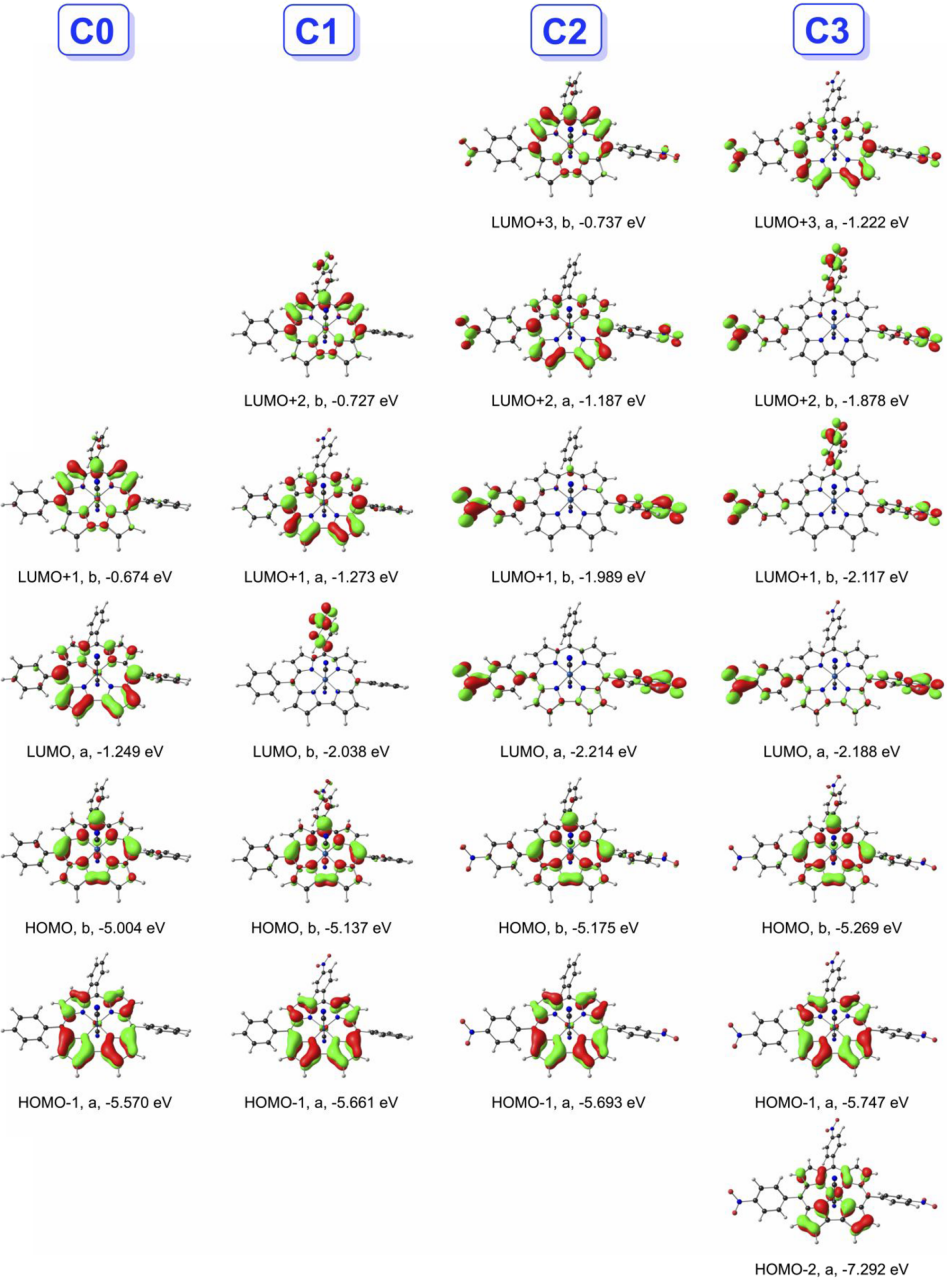
Selected CAMY-B3LYP frontier MOs of dianions **C0**–**C3**, with *C*_2_ irreps (a, b) and orbital energies in eV.

The experimental data were modeled with state-of-the-art DFT and range-separated TDDFT calculations on the four species depicted in Scheme 1. For complexes **C1**–**C3**, the nitrophenyl-based LUMOs are considerably below the corrole-based LUMO, resulting in dramatically lower HOMO–LUMO gaps relative to those of the unadorned TPC complex, **C0**, as shown in Figure 4. Paralleling experimental measurements, the lowest DFT HOMO–LUMO gap was found for the 5,15-bis(*p*-nitrophenyl) complex with an unsubstituted 10-phenyl group, **C2**, closely followed by the tris(*p-*nitrophenyl) complex, **C3**. A slightly higher HOMO–LUMO gap is predicted for 10-nitrophenyl complex **C1** with unsubstituted 5,15-phenyl groups, while a much higher HOMO–LUMO gap, understandably, is found for unadorned TPC complex **C0**. In other words, the HOMO–LUMO gaps will follow the order: **C2** < **C3** < **C1** < **C0**. CAMY-B3LYP TDDFT calculations assign the lowest-energy absorption of each system to an overwhelming HOMO-to-LUMO transition. Understandably, the TDDFT transition energies (Table 2 and Figures 5 and 6) mirror the order of Kohn–Sham HOMO–LUMO gaps.

**Table 2:** CAMY-B3LYP-D3/STO-TZ2P TDDFT Results, Including Wavelengths (λ), Oscillator Strengths (*f*), MO Compositions, and Symmetries

*E* (eV)	λ (nm)^a^	*f*	MO composition^b^			state symmetry
			weight (%)	from	to	
***Complex C0***						
1.88	659.2 **(a)**	0.56	94.6	HOMO	LUMO	B
			3.5	HOMO–1	LUMO + 1	B
2.26	548.0 **(b)**	0.03	58.6	HOMO–1	LUMO	A
			39.2	HOMO	LUMO + 1	A
2.58	481.2 **(c)**	1.58	58.5	HOMO	LUMO + 1	A
			39.2	HOMO–1	LUMO	A
2.99	414.6 (d)	1.04	91.7	HOMO–1	LUMO + 1	B
			3.5	HOMO	LUMO	B
***Complex C1***						
1.64	758.0 **(e)**	0.89	94.8	HOMO	LUMO	A
			2.5	HOMO	LUMO + 2	A
1.96	632.9 **(f)**	0.34	84.0	HOMO	LUMO + 1	B
			8.9	HOMO–1	LUMO	B
			5.1	HOMO–1	LUMO + 2	B
2.35	527.5 **(g)**	0.34	84.3	HOMO–1	LUMO	B
			11.5	HOMO	LUMO + 1	B
2.37	523.5 **(h)**	0.36	74.9	HOMO–1	LUMO + 1	A
			22.0	HOMO	LUMO + 2	A
2.71	457.8 **(i)**	0.76	72.8	HOMO	LUMO + 2	A
			22.9	HOMO–1	LUMO + 1	A
3.11	398.4 **(j)**	0.81	86.7	HOMO–1	LUMO + 2	B
			4.8	HOMO–1	LUMO	B
*Complex **C2***						
1.48	839.7 **(k)**	1.24	89.5	HOMO	LUMO	B
			7.9	HOMO	LUMO + 2	B
1.82	679.4	0.01	75.1	HOMO	LUMO + 1	A
			13.3	HOMO–1	LUMO	A
			5.3	HOMO	LUMO + 3	A
2.01	616.5 **(l)**	0.36	69.3	HOMO–1	LUMO	A
			19.6	HOMO	LUMO + 1	A
			8.8	HOMO–1	LUMO + 2	A
2.30	539.9 **(m)**	0.31	43.8	HOMO–1	LUMO + 1	B
			40.4	HOMO	LUMO + 2	B
			7.7	HOMO–1	LUMO + 3	B
			5.3	HOMO	LUMO	B
2.41	514.4 **(n)**	0.12	50.5	HOMO–1	LUMO + 1	B
			43.8	HOMO	LUMO + 2	B
2.63	471.3 **(o)**	0.25	77.5	HOMO	LUMO + 3	A
			9.0	HOMO–1	LUMO	A
			7.8	HOMO–1	LUMO + 2	A
2.84	436.2 **(p)**	0.90	76.0	HOMO–1	LUMO + 2	A
			13.8	HOMO	LUMO + 3	A
			6.5	HOMO–1	LUMO	A
3.11	398.9 **(q)**	0.56	86.0	HOMO–1	LUMO + 3	B
			5.1	HOMO	LUMO + 2	B
***Complex C3***						
1.59	778.4 **(r)**	1.04	86.4	HOMO	LUMO	B
			10.1	HOMO	LUMO + 3	B
1.74	714.1 **(s)**	0.42	88.6	HOMO	LUMO + 1	A
			4.6	HOMO	LUMO + 4	A
2.05	604.8 **(t)**	0.59	63.7	HOMO–1	LUMO	A
			17.0	HOMO	LUMO + 2	A
			12.9	HOMO–1	LUMO + 3	A
2.12	583.8	0.00	79.3	HOMO	LUMO + 2	A
			14.7	HOMO–1	LUMO	A
2.26	549.2 **(u)**	0.46	69.5	HOMO–1	LUMO + 1	B
			14.6	HOMO	LUMO + 3	B
			7.9	HOMO–1	LUMO + 4	B
2.46	504.9 **(v)**	0.06	64.1	HOMO	LUMO + 3	B
			20.5	HOMO–1	LUMO + 1	B
			6.4	HOMO–1	LUMO + 2	B
			5.7	HOMO	LUMO	B
2.61	475.2	0.01	90.7	HOMO–1	LUMO + 2	B
			4.0	HOMO	LUMO + 3	B
2.72	455.6	0.03	65.6	HOMO	LUMO + 4	A
			16.5	HOMO–1	LUMO + 3	A
			11.5	HOMO–1	LUMO	A
2.88	430.8 **(w)**	0.76	65.3	HOMO–1	LUMO + 3	A
			25.0	HOMO	LUMO + 4	A
3.17	391.4 **(x)**	0.50	84.0	HOMO–1	LUMO + 4	B
			4.7	HOMO–1	LUMO + 1	B
			4.3	HOMO	LUMO + 3	B

a The letters in bold in the second column refer to peak labels in Figure 5.

b The MOs are visually depicted in Figure 6.

For **C0**–**C3**, the largest redshift of the lowest-energy absorption is observed for dicyanido-cobalt 5,15-bis(*p*-nitrophenyl)-10-phenylcorrole, i.e., complex **C2** (expt 826 nm in Table 1 and Figure 1; calc peak k at 839.7 nm in Figure 5 and Table 2) The second largest redshift for the NIR transition, both experimentally and theoretically, is exhibited by complex **C3** (expt 798 nm in Table 1 and Figure 1; calc peak r at 778.4 nm in Figure 5 and Table 2) The third spot, experimentally, is occupied by the 5,15-dimesityl-10-(*p*-nitrophenyl)corrole complex (expt 732 nm^19^), which has been modeled here as complex **C1** (calc peak e at 758 nm in Figure 5 and Table 2). The least red-shifted complex is that of *meso*-tris(*p*-*t*-butylphenyl)corrole (expt 696 nm^19^), which has been modeled here with TPC, i.e., complex **C0** (calc peak a at 659 nm in Figure 5 and Table 2).

The calculations also permit plausible assignments of the remainder of the optical spectra. The experimentally studied species all exhibit an absorption in the 575–595 nm range, which appears as a shoulder in the majority of cases but as a distinct peak for {Co[T*p*NO_2_PC](CN)_2_}^2–^ (**C3**) (Figure 1). This feature appears to correspond to essentially a (HOMO–1)-to-nitrophenyl transition (peak l at 616.5 nm for **C2** and peak t at 604.8 nm for **C3**) in Figure 5 and Table 2, where HOMO–1 can be identified with the corrole analogue of the Gouterman a_1u_ orbital of porphyrins. The relatively higher intensity of the feature for **C3** appears to reflect additional charge transfer character mixing into the overall composition of the transition, as a result of the presence of the 10-nitrophenyl group. Finally, a relatively normal Gouterman-type four-orbital composition is indicated for the intense Soret-like features under 500 nm, i.e., peaks i and j for **C1**, peaks o–q for **C2**, and peaks w and x for **C3** (see Figure 5 and Table 2).^41^

Overall, the calculated transition energies are in impressive, semiquantitative agreement with experimental absorption maxima, within the resolution of solution-phase spectroscopic measurements and allow for multiple conformations and details of solvation that we have not accounted for in our calculations. The agreement is all the more remarkable in that, for **C1**–**C3**, the lower-energy transitions largely involve corrole-to-nitrophenyl charge transfer character. TDDFT calculations routinely struggle with predicting the energetics of charge transfer transitions. Key to our success in the present study has been our earlier groundwork on hyperporphyrin systems,^10,42^ which established the importance of using an appropriate solvation model and a range-separated functional such as CAMY-B3LYP that provides improved description for charge transfer transitions.^35−37^ It will indeed be interesting to see how well the present methods perform vis-à-vis other anionic hyperporphyrin systems such as *O*-deprotonated *meso*-tetrakis(*p*-hydroxyphenyl)corrole.^43,44^

## Discussion

The UV–vis-NIR spectra of dicyanidocobalt 5,15-bis(*p*-nitrophenyl) corroles, where the 10-position can vary, may be viewed as paradigms of inverse hyper spectra, with clean macrocycle-to-substituent charge-transfer transitions in the near-infrared. Such transitions reflect a clean LUMO switch in these systems (relative to other *meso*-triarylcorroles and tetraarylporphyrins), from macrocycle- to *meso*-aryl-based, as a result of the *relatively weak electronic coupling* between the macrocycle and the significantly twisted (i.e., out-of-plane) aryl substituents. These systems may be contrasted with β-formyl-,^45^ dicyanovinyl- and dicyanobutadienyl- metallocorroles^46,47^ in which strong NIR absorptions primarily reflect an extension of the corrole’s conjugation, with varying contributions of corrole-to-substituent charge transfer character.

It is interesting to reflect on the role of the metal center in engendering inverse hypercorrole spectra for *meso*-nitrophenyl-appended corroles. Some of us have suggested that an innocent corrole macrocycle is critical.^19^ For example, whereas neutral Cu[T*p*NO_2_PC] (the Cu analogue of **C3**), in which the corrole is thought to be noninnocent,^48−53^ does not exhibit much of a hypercorrole spectrum (in the form of strong NIR absorption), the innocent^54,55^ anionic species {Cu[T*p*NO_2_PC]}^−^ exhibits a pronounced inverse hypercorrole spectrum similar to **C3**. Yet, an innocent *meso*-nitrophenyl-appended corrole, though possibly necessary for a pronounced inverse hypercorrole spectrum, does not guarantee one. Thus, innocent monocyanido analogues of the dicyanido cobalt corroles studied here do not exhibit an equally pronounced inverse hypercorrole effect. Nor, for that matter, do *p*-nitrophenyl-appended cobalt(III)-triphenylphosphine corroles.^56^ In the same vein, *meso*-*p*-nitrophenyl groups by themselves do not appear to elicit much of a hyperporphyrin effect in charge-neutral metalloporphyrins^57^ (although extending the conjugation with *meso*-*p*-nitrophenylethynyl groups does engender large spectral redshifts^58^). The dianionic character of the dicyanido complexes studied here and the sizable, negative formal charge on the cobalt (in spite of the + III oxidation state^59^) play a critical role in engendering the observed hypercorrole spectra.

The importance of the overall negative charge on the metal–corrole fragment and of anionic axial ligands immediately suggests applications of nitrophenyl-appended porphyrins and corroles as anion sensors. A handful of applications to the selective sensing of neutral ligands and heavy metal cations (such as Hg^2+^^60^ and Ru^3+^^61^) have already been reported in the literature; these systems, however, exhibit only modest, if any, NIR absorption and are at best viewed as incipient inverse hypercorroles. With the concept of an “inverse hypercorrole” authenticated both experimentally and theoretically as a result of this work, there is clearly considerable room for creativity in the design of new anion chemosensors. In the same vein, biocompatible inverse hyperporphyrins and hypercorroles, on account of their NIR emission, may lend themselves to applications in photomedicine, as new dyes for photodynamic and photothermal therapies and as physiological oxygen sensors.^13,14^

## Conclusions

In a 2022 *Perspective* on *The Hyperporphyrin Concept*,^6^ we noted that hyper spectra arising via macrocycle-to-*meso*-aryl charge transfer were unknown. Herein, state-of-the-art TDDFT calculations have supported the formulation of *meso-p*-nitrophenyl-appended dicyanidocobalt(III) corroles as paradigmatic “inverse hypercorroles”. The intense NIR absorptions of these corroles are ascribed to a transition from the corrole HOMO (with a porphyrin a_2u_-like shape) to a nitrophenyl-based LUMO. The hypercorrole effect, as measured by the redshift and intensity of the NIR absorption, is particularly dramatic for 5,15-bis(*p*-nitrophenyl)-substituted complexes, with *para* substituents on the 10-phenyl group exercising a modulating influence. The simplicity of inverse hypercorrole design (involving *meso*-*p*-nitrophenyl substituents and anionic axial ligands) provides an attractive alternative to the traditional approach to NIR-absorbing porphyrinoids in which the macrocycle’s π-system is extended by conjugating substituents or arene annulation. Accordingly, we harbor the hope that applications of inverse hypercorroles to areas such as anion sensing and photomedicine will emerge in relatively short order, an exciting prospect from the perspective of the present study.

## Supporting Information Available

MALDI/TOF LRMS, ESI HRMS spectra, and ^1^H NMR spectra of cobalt complexes **S1**–**S6**. Optimized coordinates of the DFT calculations (PDF)
