# Supplementary material for: Inverse Hypercorroles
Source: Inorg Chem. 2024 May 2;63(19):8739–49. doi: 10.1021/acs.inorgchem.4c00344 (PMC11094798; doi:10.1021/acs.inorgchem.4c00344)
Supplement: Supplementary file 1 — ic4c00344_si_001.pdf [file ic4c00344_si_001.pdf]

Inverse Hypercorroles

W. Ryan Osterloh,[a] Nicolas Desbois,[a] Jeanet Conradie,[b,c] Claude P. Gros,[a] Karl M. Kadish[d] and Abhik Ghosh*,[c]

[a] ICMUB (UMR CNRS 6302), Université de Bourgogne, 9, Avenue A. Savary, BP 47870, 21078 Dijon, Cedex, France
[b] Department of Chemistry, UiT – The Arctic University of Norway, N-9037 Tromsø, Norway;
[c] Department of Chemistry, University of the Free State, 9300 Bloemfontein, Republic of South Africa.
[d] Department of Chemistry, University of Houston, Houston, Texas 77204-5003, USA.

Correspondence: Email: abhik.ghosh@uit.no (AG)

Supporting information

Table of Contents

**Figure S1**	MALDI/TOF LRMS and ESI HRMS spectra of metallocorrole **S2.**	S2
**Figure S2**	^{1}H NMR (top, 500 MHz) and ^{19}F NMR (down, 470 MHz) spectra of metallocorrole **S2** in $CDCl_3 + NH_3$.	S3
**Figure S3**	MALDI/TOF LRMS and ESI HRMS spectra of metallocorrole **S3.**	S4
**Figure S4**	^{1}H NMR (500 MHz) spectrum of metallocorrole **S3** in $CDCl_3 + NH_3$.	S5
**Figure S5**	MALDI/TOF LRMS and ESI HRMS spectra of the free-base corrole ligand of **S5**.	S6
**Figure S6**	^{1}H NMR (500 MHz) spectrum of the free base corrole of **S5** in $CDCl_3 + NH_3$.	S7
**Figure S7**	MALDI/TOF LRMS and ESI HRMS spectra of metallocorrole **S5**.	S8
**Figure S8**	^{1}H NMR (400 MHz) spectrum of metallocorrole **S5** in $CDCl_3 + NH_3$.	S9
**Figure S9**	MALDI/TOF LRMS and ESI HRMS spectra of the free-base corrole ligand of **S6**.	S10
**Figure S10**	^{1}H NMR (500 MHz) spectrum of the free-base corrole ligand of **S6** in $CDCl_3 + NH_3$.	S11
**Figure S11**	MALDI/TOF LRMS and ESI HRMS spectra of metallocorrole **S6**.	S12
**Figure S12**	^{1}H NMR (500 MHz) spectrum of metallocorrole **S6** in $CDCl_3 + NH_3$.	S13
**Optimized Cartesian coordinates (Å)**		S14
1. **C0**: $\{Co[TPC](CN)_2\}^{2-}$		S14
2. **C1**: $\{Co[(5,15\text{-}P)(10\text{-}p NO_2P)C](CN)_2\}^{2-}$		S16
3. **C2**: $\{Co[(5,15\text{-}p NO_2P)(10\text{-}P)C](CN)_2\}^{2-}$		S18
4. **C3**: $\{Co[Tp NO_2PC](CN)_2\}^{2-}$		S20

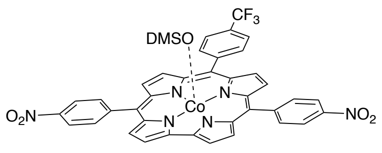

Chemical Formula: $C_{40}H_{26}CoF_3N_6O_5S$
Exact Mass: 818.0969
Molecular Weight: 818.6734
$[M\text{-}DMSO]^{+\cdot}$: Chemical Formula: $C_{38}H_{20}CoF_3N_6O_4$
Exact Mass: 740.0830

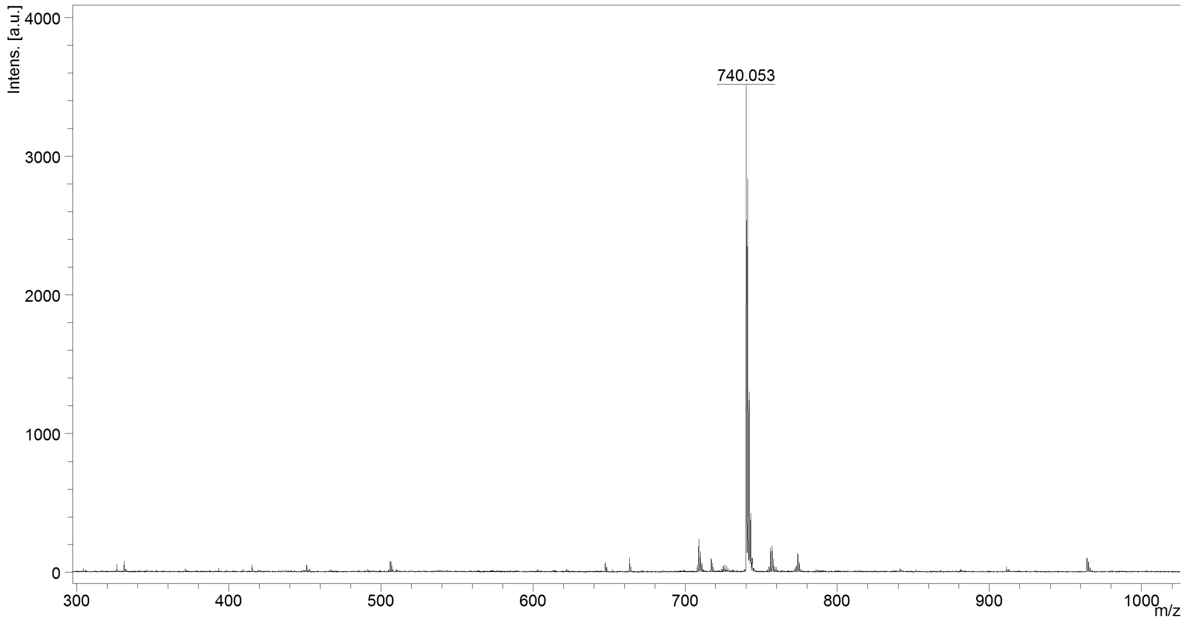

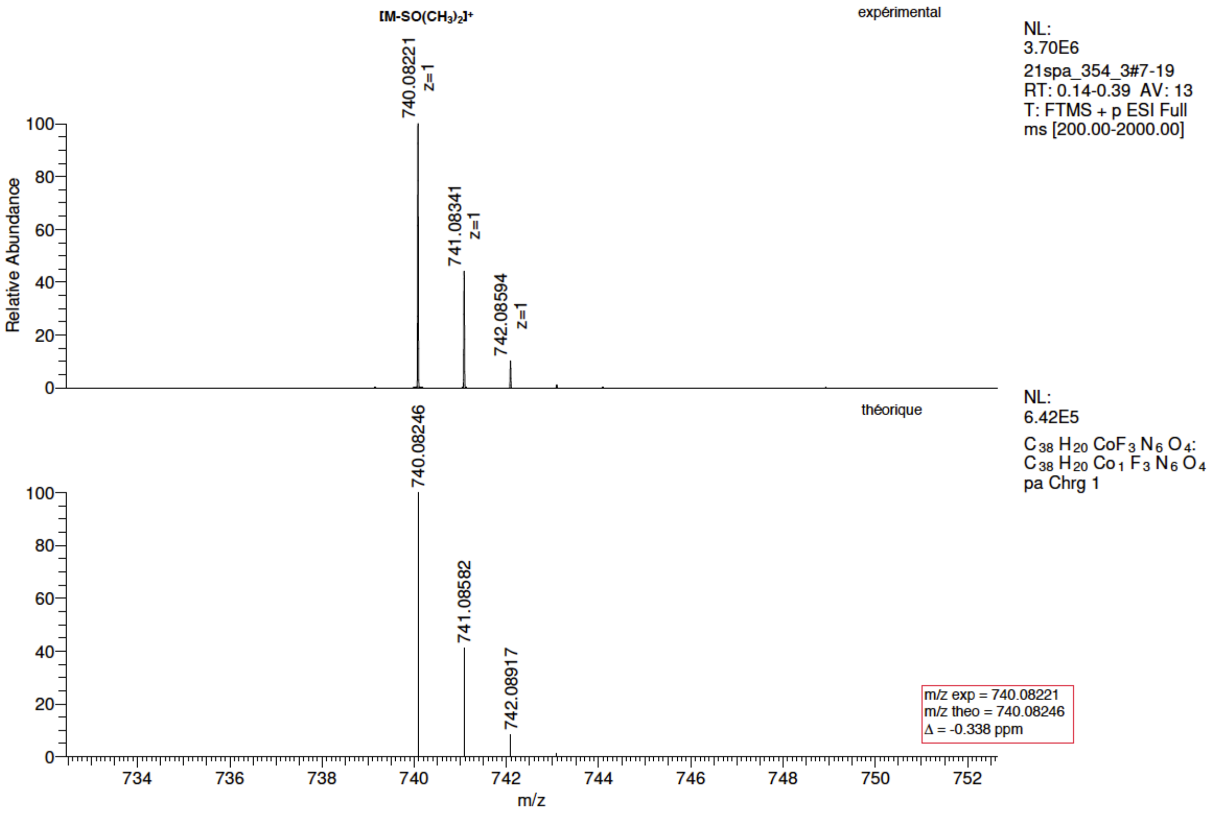

Figure S1. MALDI/TOF LRMS and ESI HRMS spectra of metallocorrole **S2**.

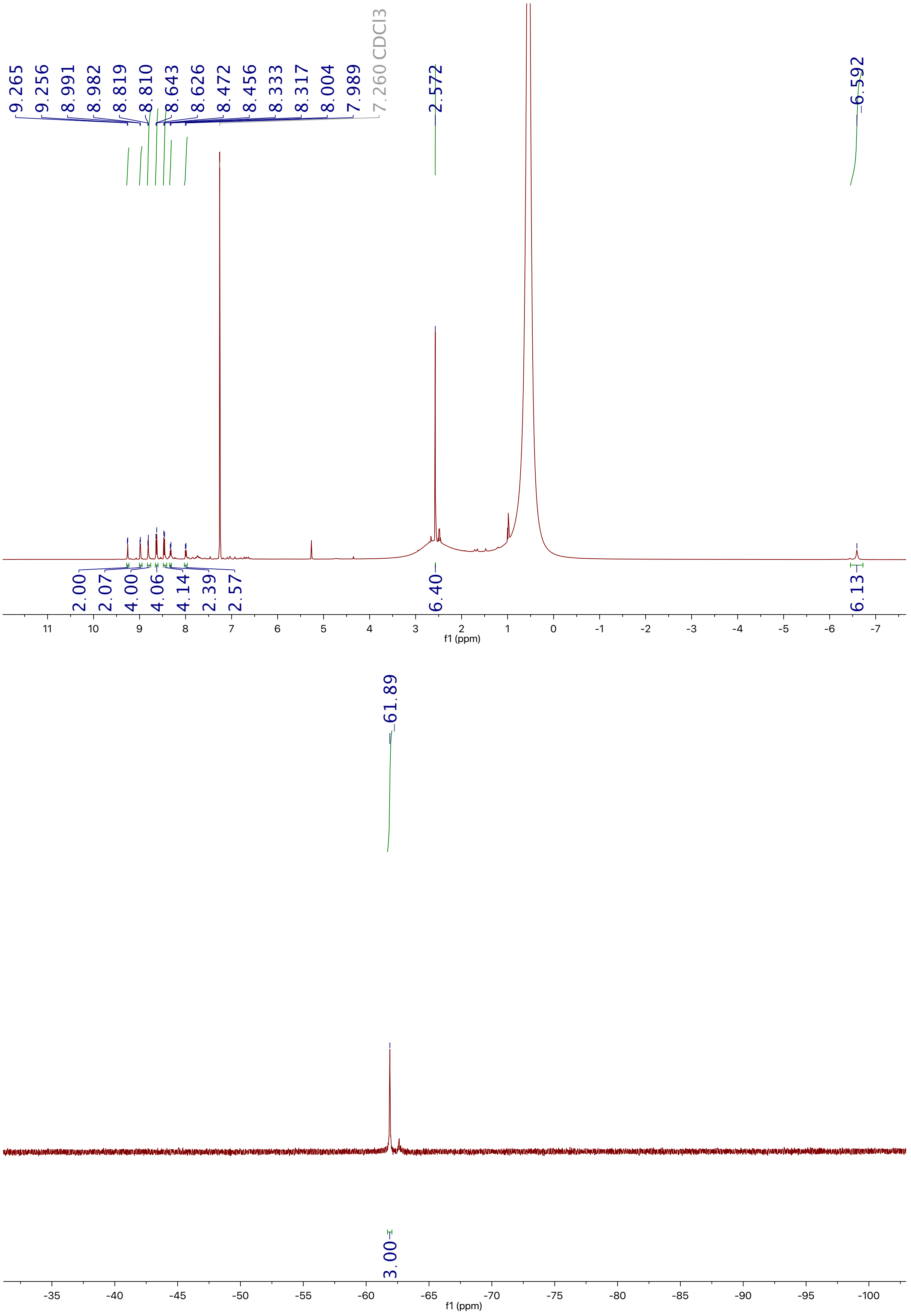

Figure S2: ^{1}H NMR (top, 500 MHz) and ^{19}F NMR (down, 470 MHz) spectra of metallocorrole **S2** in $CDCl_3$ + NH_3.

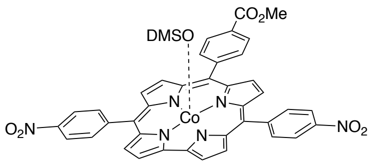

Chemical Formula: $C_{42}H_{26}CoF_9N_4OS$
Exact Mass: 864.1016
Molecular Weight: 864.6758
[M-DMSO]$^{+\cdot}$: Chemical Formula: $C_{39}H_{23}CoN_6O_6$
Exact Mass: 730.1011

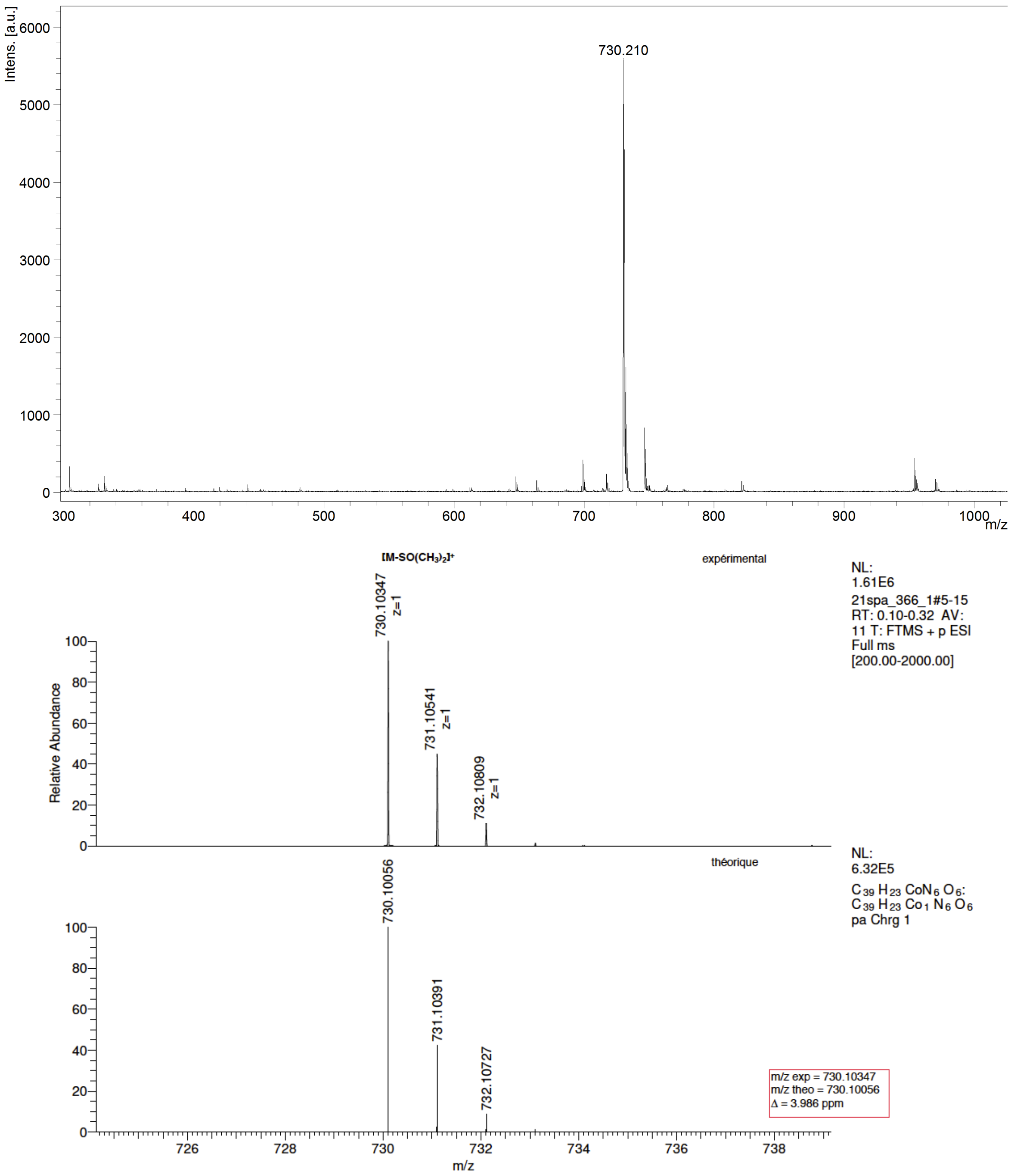

Figure S3. MALDI/TOF LRMS and ESI HRMS spectra of metallocorrole **S3**.

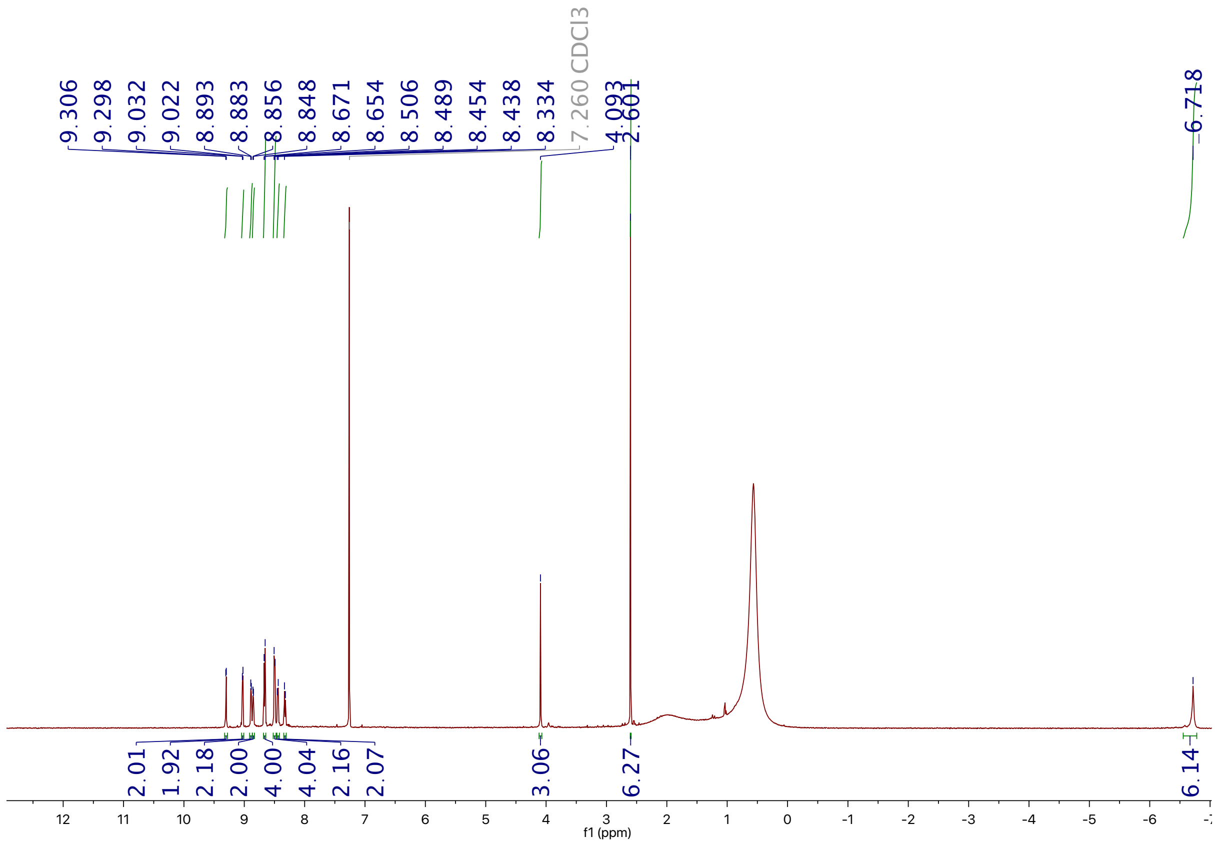

Figure S4. ¹H NMR (500 MHz) spectrum of metallocorrole **S3** in $CDCl_3 + NH_3$.

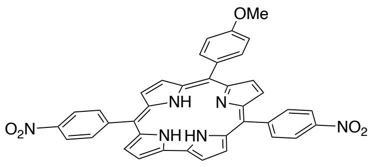

Chemical Formula: $C_{38}H_{26}N_6O_5$
Exact Mass: 646.1965
Molecular Weight: 646.6630

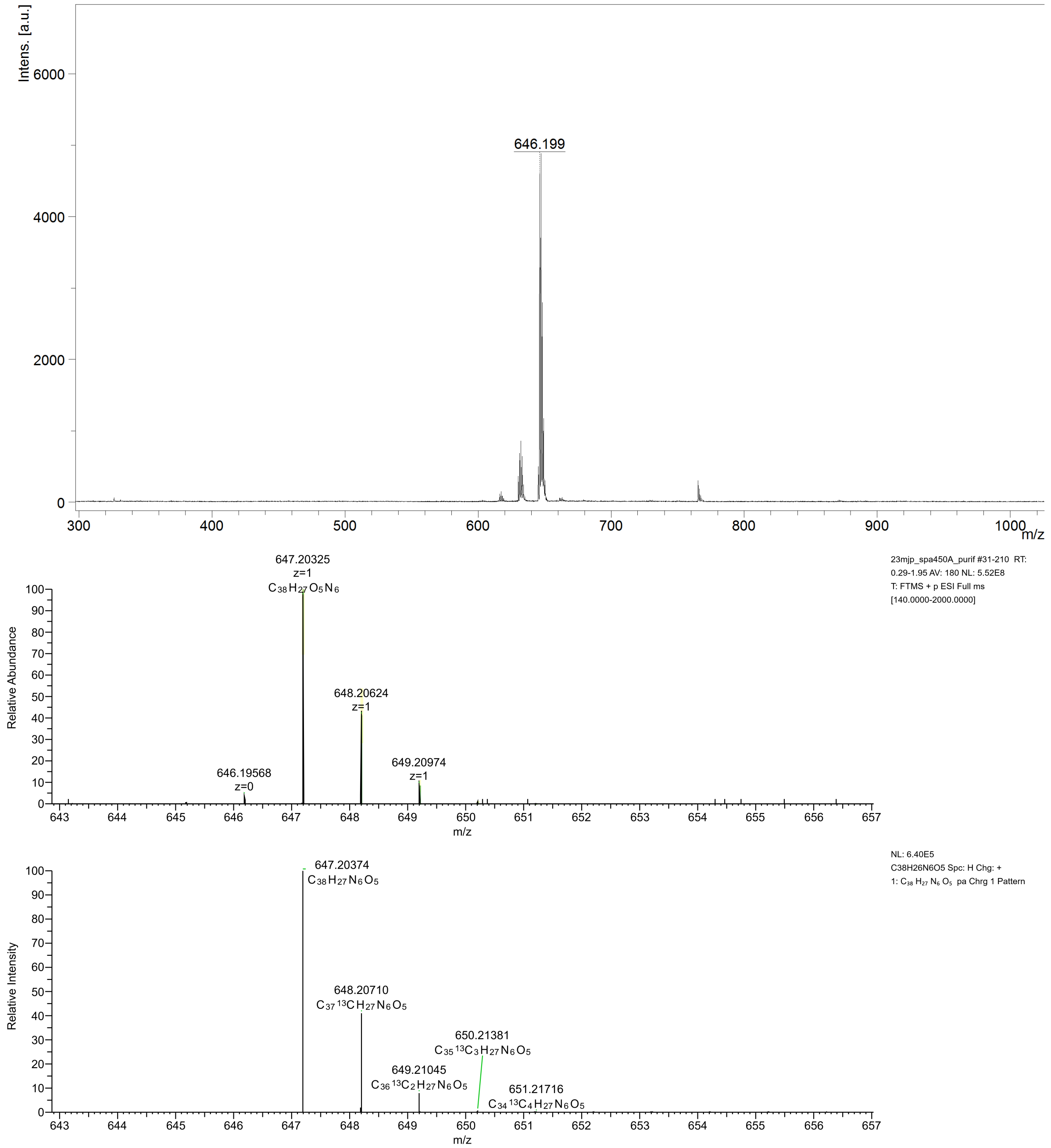

Peak Mass	Display Formula	RDB	Delta [ppm]	Theo. mass	Rank	Pattern Cov. [%]	MSMS Matched Fragm...
647.20325	$C_{38}H_{27}O_5N_6$	28.50	-0.77	647.20374	1	100	(Collection)

Figure S5. MALDI/TOF LRMS and ESI HRMS spectra of the free-base corrole ligand of **S5**.

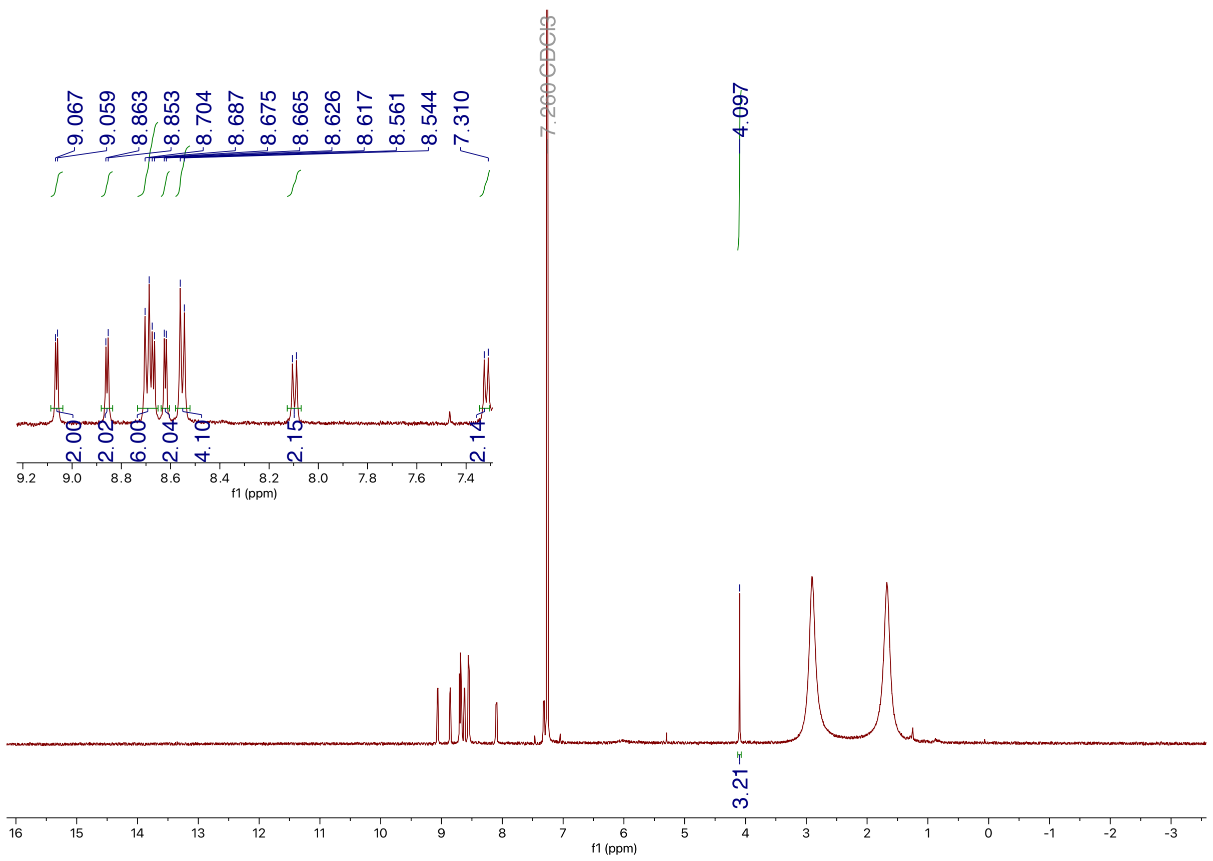

Figure S6. ^{1}H NMR (500 MHz) spectrum of the free-base corrole ligand of **S5** in $CDCl_3$ + NH_3.

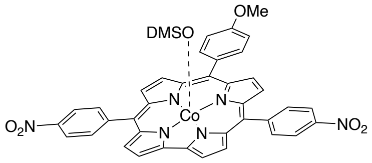

Chemical Formula: $C_{40}H_{29}CoN_6O_6S$
Exact Mass: 780.1201
Molecular Weight: 780.7012
$[M\text{-}DMSO]^{+\cdot}$: Chemical Formula: $C_{38}H_{23}CoN_6O_5$
Exact Mass: 702.1056

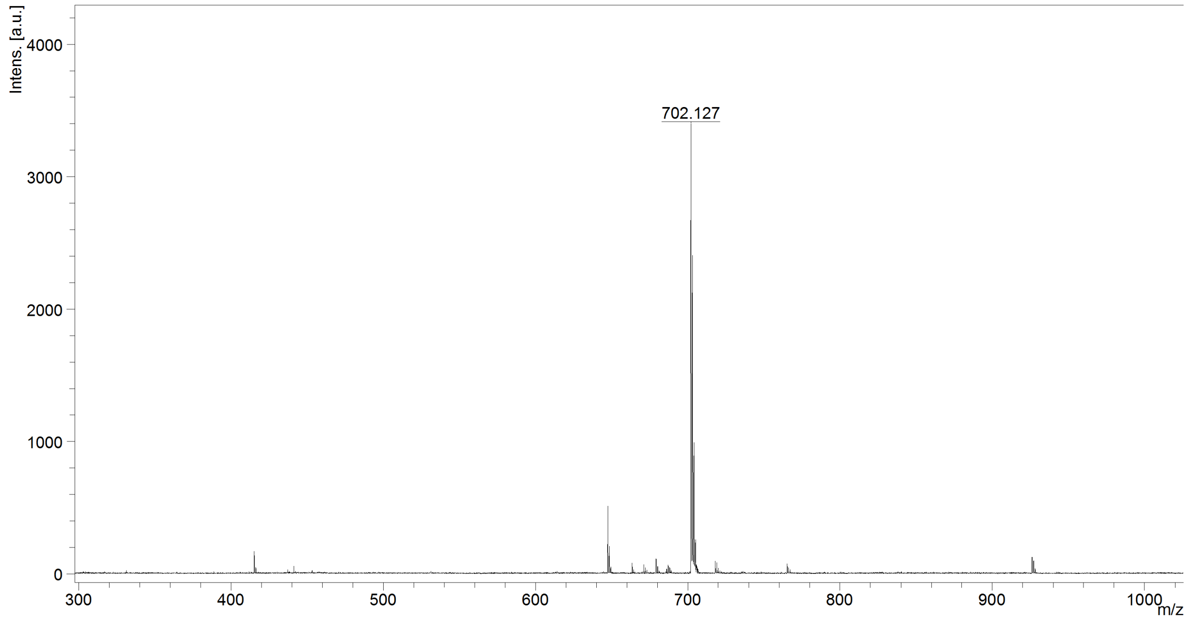

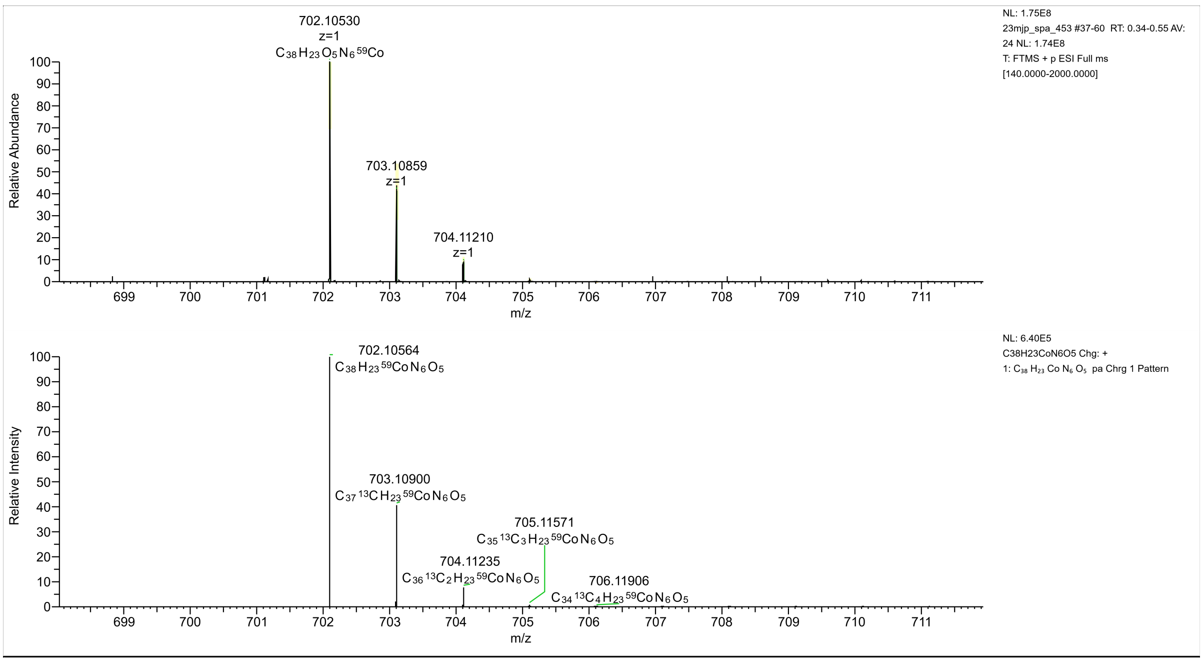

Peak Mass	Display Formula	RDB	Delta [ppm]	Theo. mass	Rank	Pattern Cov. [%]	MSMS Matched Fragm...
702.10530	$C_{38}H_{23}O_5N_6{}^{59}Co$	30.50	-0.49	702.10564	1	99.89	(Collection)

Figure S7. MALDI/TOF LRMS and ESI HRMS spectra of metallocorrole **S5**.

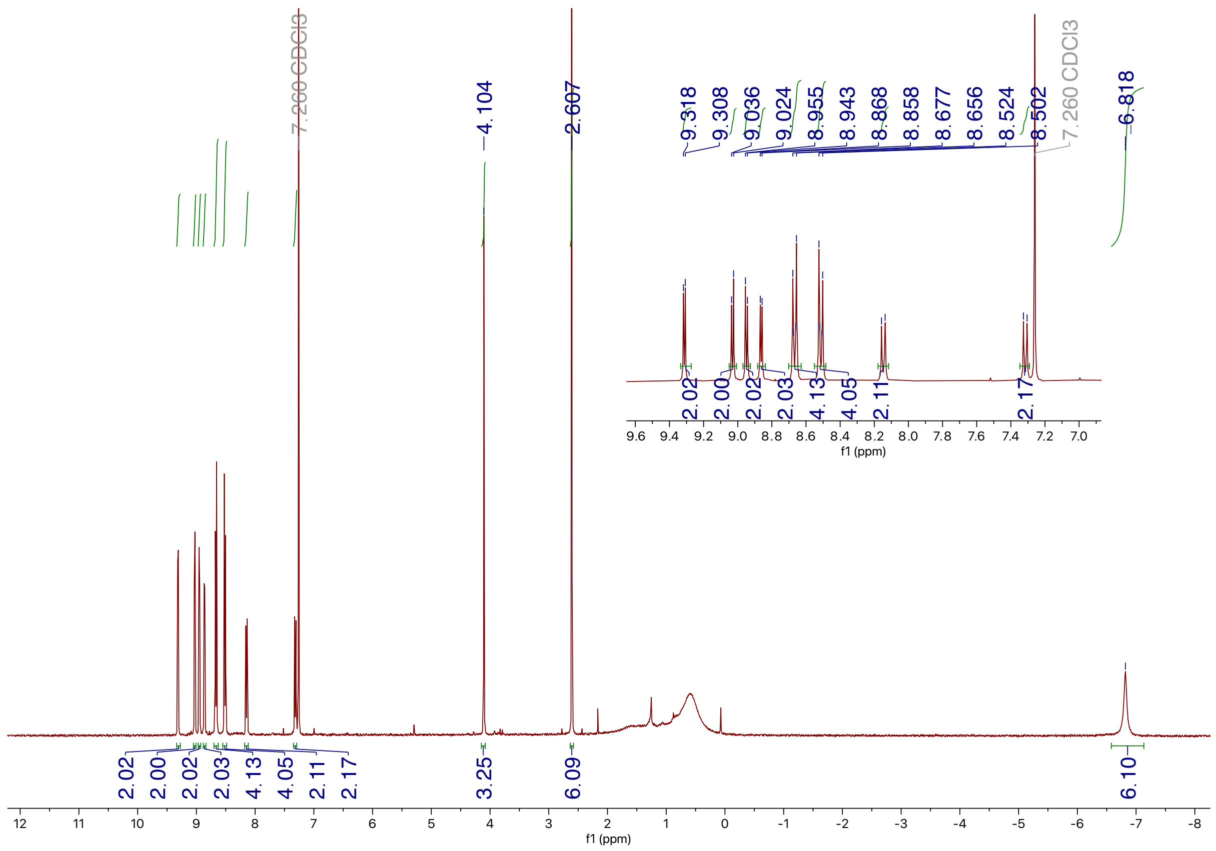

Figure S8. ^{1}H NMR (400 MHz) spectrum of metallocorrole **S5** in $CDCl_3$ + NH_3.

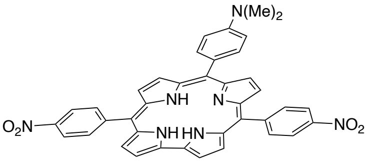

Chemical Formula: $C_{39}H_{29}N_7O_4$
Exact Mass: 659.2281
Molecular Weight: 659.7060

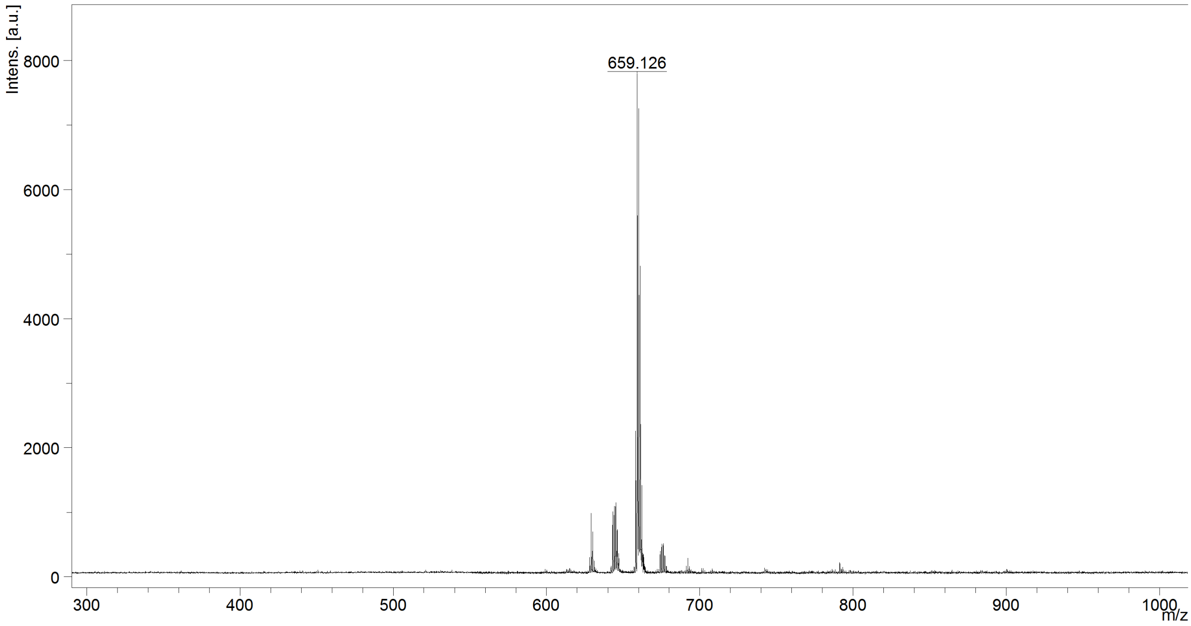

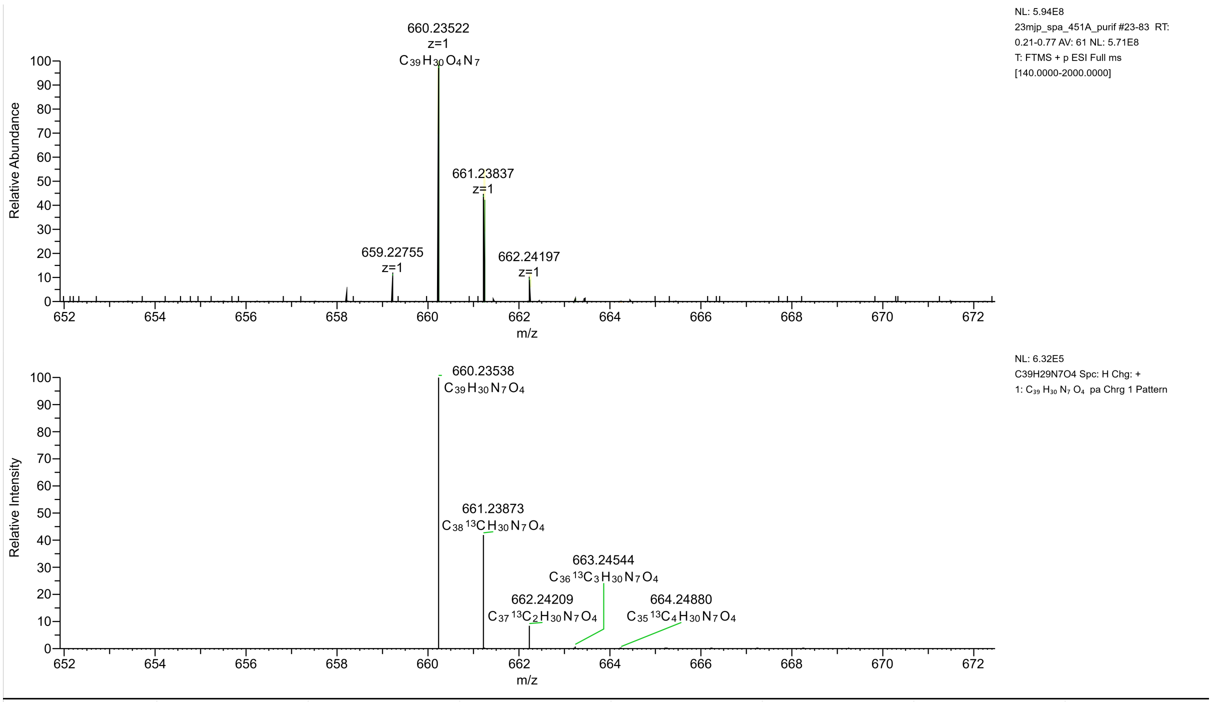

Peak Mass	Display Formula	RDB	Delta [ppm]	Theo. mass	Rank	Pattern Cov. [%]	MSMS Matched Fragm...
660.23522	$C_{39}H_{30}O_4N_7$	28.50	-0.25	660.23538	1	99.9	(Collection)

Figure S9. MALDI/TOF LRMS and ESI HRMS spectra of the free-base corrole ligand of **S6**.

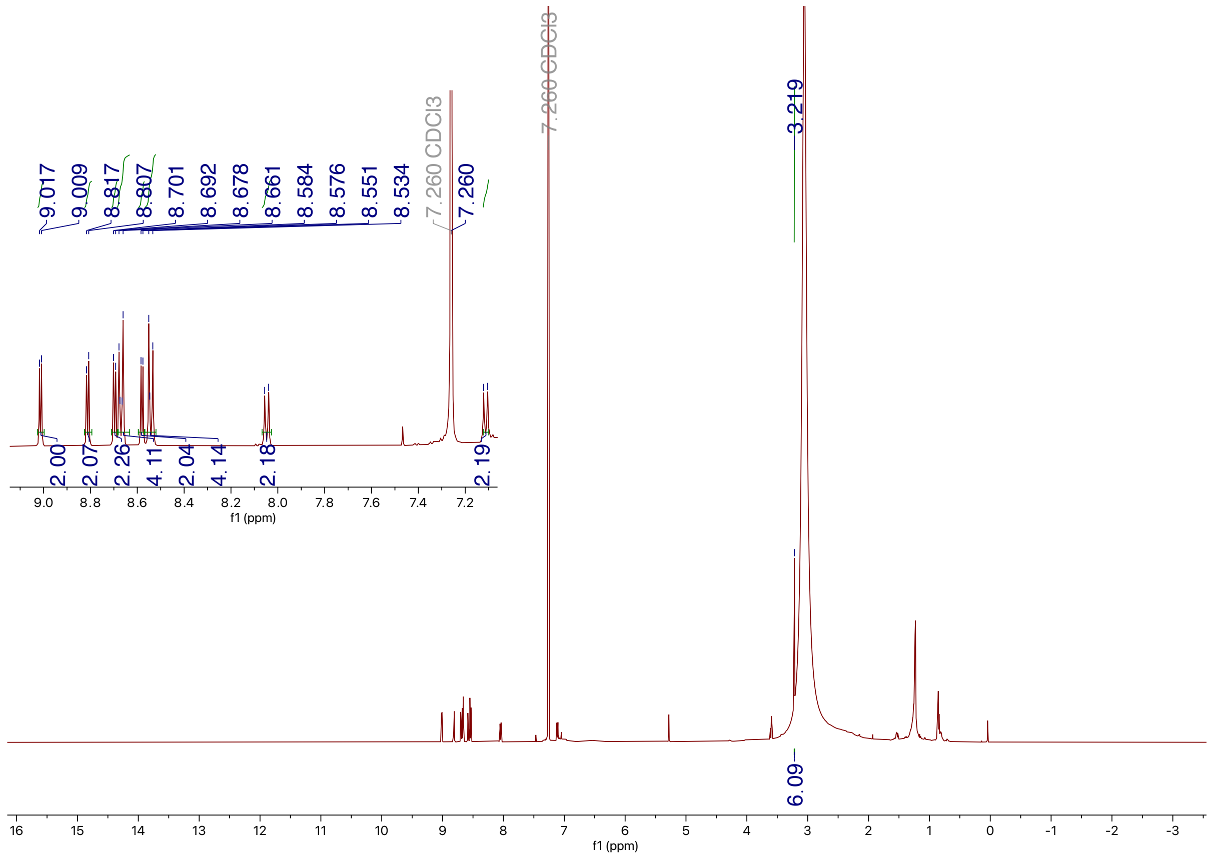

Figure S10. ^{1}H NMR (500 MHz) spectrum of the free-base corrole ligand of **S6** in $CDCl_3$ + NH_3.

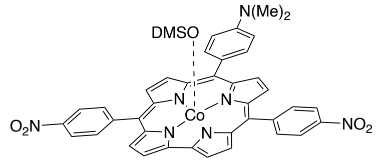

Chemical Formula: $C_{41}H_{32}CoN_7O_5S$
Exact Mass: 793.1518
Molecular Weight: 793.7442
$[M\text{-}DMSO]^{+\cdot}$: Chemical Formula: $C_{39}H_{26}CoN_7O_4$
Exact Mass: 715.1373

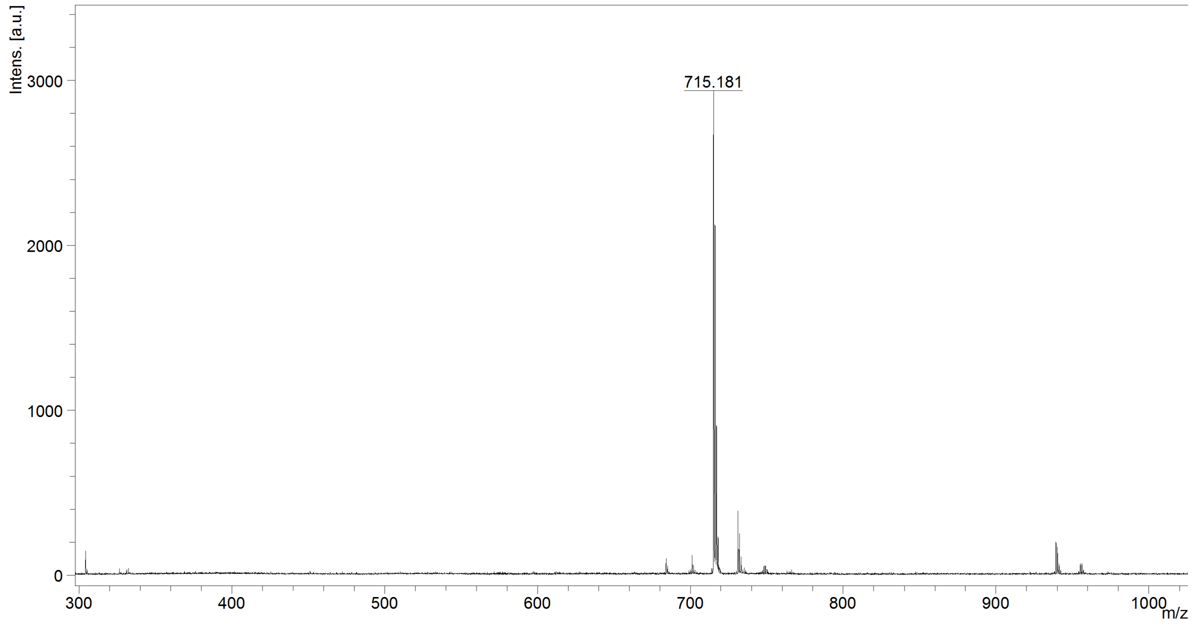

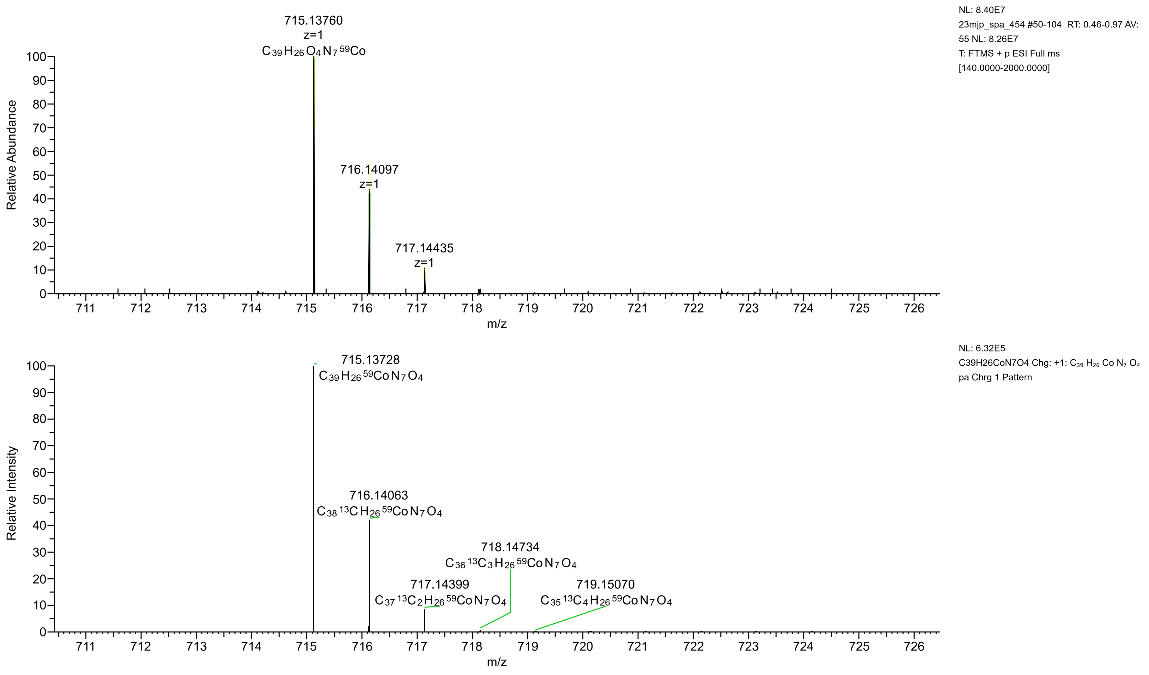

Peak Mass	Display Formula	RDB	Delta [ppm]	Theo. mass	Rank	Pattern Cov. [%]	MSMS Matched Fragm...
715.13760	$C_{39}H_{26}O_4N_7{}^{59}Co$	30.50	0.44	715.13728	1	100	(Collection)

Figure S11. MALDI/TOF LRMS and ESI HRMS spectra of metallocorrole **S6**.

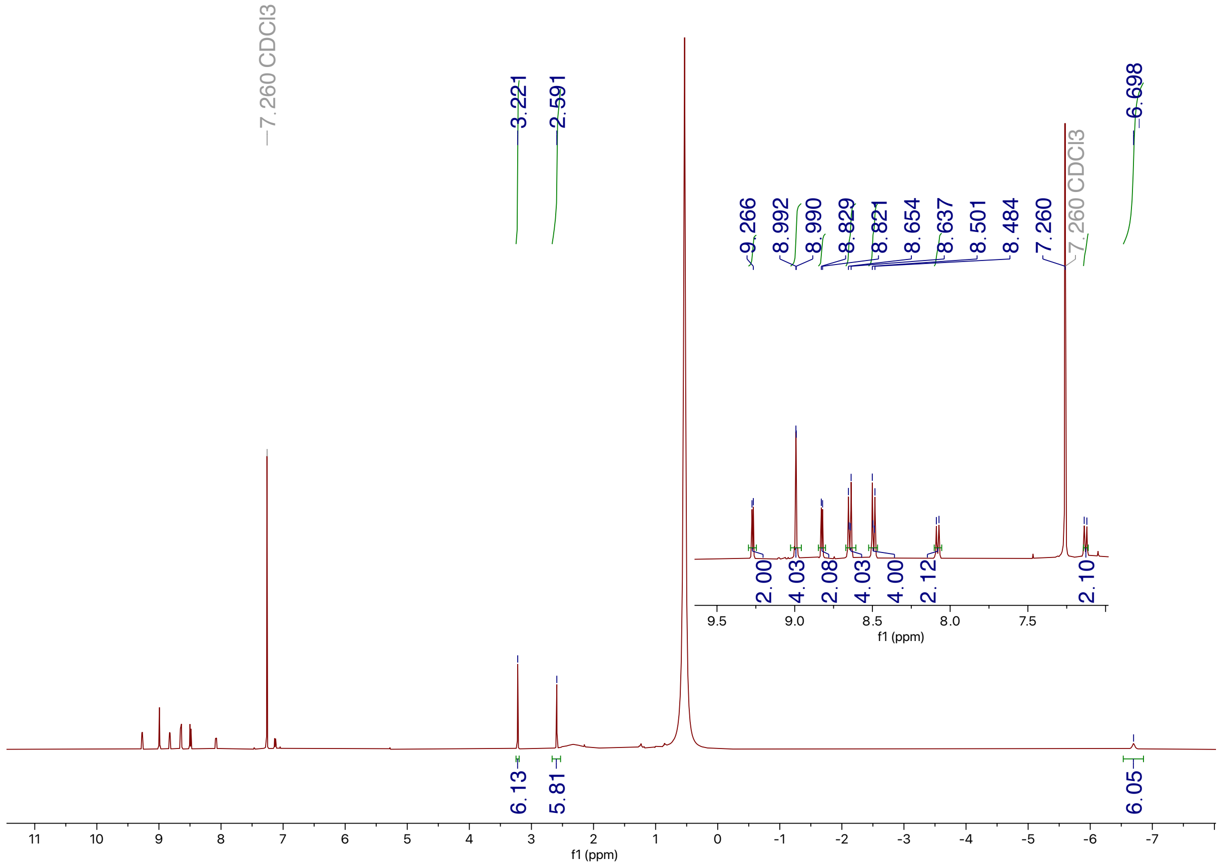

Figure S12. ^{1}H NMR (500 MHz) spectrum of metallocorrole **S6** in $CDCl_3$ + NH_3.

Optimized OLYP/ZORA-STO-TZ2P Cartesian coordinates (Å)

C0: {Co[TPC](CN)$_2$}$^{2-}$

Co	0.000000000	0.000000000	-0.627865000
C	0.000000000	0.000000000	2.672356000
C	0.000000000	0.000000000	4.157836000
C	0.000000000	0.000000000	6.982179000
C	0.299131000	-1.166453000	4.881624000
C	0.304543000	-1.167479000	6.276454000
C	0.538758000	1.812642000	-0.616994000
C	0.681665000	-0.200389000	-3.312385000
C	1.193687000	-0.358889000	2.009390000
C	1.729692000	-0.469898000	-4.238651000
C	2.449801000	-0.703452000	2.630993000
C	2.488639000	-0.712444000	-2.096311000
C	2.673987000	-0.736777000	0.370284000
C	2.858529000	-0.782254000	-3.484531000
C	3.243135000	-0.891197000	-0.912517000
C	3.350385000	-0.945292000	1.626782000
C	4.677937000	-1.240784000	-1.029991000
C	5.088463000	-2.360770000	-1.773597000
C	5.669850000	-0.458599000	-0.412429000
C	6.438172000	-2.694504000	-1.885143000
C	7.019696000	-0.791878000	-0.519561000
C	7.411831000	-1.913433000	-1.255770000
C	-0.299131000	1.166453000	4.881624000
C	-0.304543000	1.167479000	6.276454000
C	-0.538758000	-1.812642000	-0.616994000
C	-0.681665000	0.200389000	-3.312385000
C	-1.193687000	0.358889000	2.009390000
C	-1.729692000	0.469898000	-4.238651000
C	-2.449801000	0.703452000	2.630993000
C	-2.488639000	0.712444000	-2.096311000
C	-2.673987000	0.736777000	0.370284000
C	-2.858529000	0.782254000	-3.484531000
C	-3.243135000	0.891197000	-0.912517000
C	-3.350385000	0.945292000	1.626782000
C	-4.677937000	1.240784000	-1.029991000
C	-5.088463000	2.360770000	-1.773597000
C	-5.669850000	0.458599000	-0.412429000
C	-6.438172000	2.694504000	-1.885143000
C	-7.019696000	0.791878000	-0.519561000
C	-7.411831000	1.913433000	-1.255770000
H	0.000000000	0.000000000	8.069955000
H	0.527411000	-2.077701000	4.336364000
H	0.538261000	-2.084180000	6.814146000
H	1.653891000	-0.429998000	-5.318420000
H	2.621514000	-0.756903000	3.695823000
H	3.841782000	-1.034880000	-3.855671000
H	4.334489000	-2.976405000	-2.254865000

H	4.383732000	-1.241592000	1.730428000
H	5.369885000	0.419376000	0.151532000
H	6.730162000	-3.571857000	-2.458599000
H	7.768188000	-0.169741000	-0.033119000
H	8.464431000	-2.174337000	-1.340053000
H	-0.527411000	2.077701000	4.336364000
H	-0.538261000	2.084180000	6.814146000
H	-1.653891000	0.429998000	-5.318420000
H	-2.621514000	0.756903000	3.695823000
H	-3.841782000	1.034880000	-3.855671000
H	-4.334489000	2.976405000	-2.254865000
H	-4.383732000	1.241592000	1.730428000
H	-5.369885000	-0.419376000	0.151532000
H	-6.730162000	3.571857000	-2.458599000
H	-7.768188000	0.169741000	-0.033119000
H	-8.464431000	2.174337000	-1.340053000
N	0.871437000	2.936537000	-0.620248000
N	1.176400000	-0.359641000	-2.050716000
N	1.365777000	-0.393049000	0.653025000
N	-0.871437000	-2.936537000	-0.620248000
N	-1.176400000	0.359641000	-2.050716000
N	-1.365777000	0.393049000	0.653025000

C1: {Co[(5,15-P)(10-*p*NO$_2$P)C](CN)$_2$}$^{2-}$

Co	0.000000000	0.000000000	-1.194564000
C	0.000000000	0.000000000	2.114002000
C	0.000000000	0.000000000	3.579513000
C	0.000000000	0.000000000	6.391935000
C	0.230269000	1.876785000	-1.181529000
C	0.668095000	-1.007589000	4.314642000
C	0.671851000	-1.016306000	5.697042000
C	0.706595000	-0.080427000	-3.880402000
C	1.244505000	-0.154148000	1.443985000
C	1.785916000	-0.160096000	-4.807943000
C	2.549662000	-0.223896000	2.056881000
C	2.572478000	-0.264170000	-2.667059000
C	2.761444000	-0.246453000	-0.200953000
C	2.950447000	-0.267843000	-4.054749000
C	3.343855000	-0.295956000	-1.484836000
C	3.473429000	-0.296463000	1.047960000
C	4.819703000	-0.362742000	-1.609691000
C	5.432164000	-1.396585000	-2.337013000
C	5.639528000	0.618242000	-1.027399000
C	6.819155000	-1.451873000	-2.472473000
C	7.026478000	0.564006000	-1.160467000
C	7.623395000	-0.472186000	-1.883701000
C	-0.230269000	-1.876785000	-1.181529000
C	-0.668095000	1.007589000	4.314642000
C	-0.671851000	1.016306000	5.697042000
C	-0.706595000	0.080427000	-3.880402000
C	-1.244505000	0.154148000	1.443985000
C	-1.785916000	0.160096000	-4.807943000
C	-2.549662000	0.223896000	2.056881000
C	-2.572478000	0.264170000	-2.667059000
C	-2.761444000	0.246453000	-0.200953000
C	-2.950447000	0.267843000	-4.054749000
C	-3.343855000	0.295956000	-1.484836000
C	-3.473429000	0.296463000	1.047960000
C	-4.819703000	0.362742000	-1.609691000
C	-5.432164000	1.396585000	-2.337013000
C	-5.639528000	-0.618242000	-1.027399000
C	-6.819155000	1.451873000	-2.472473000
C	-7.026478000	-0.564006000	-1.160467000
C	-7.623395000	0.472186000	-1.883701000
H	1.163244000	-1.808604000	3.777315000
H	1.166342000	-1.806109000	6.249880000
H	1.702909000	-0.133019000	-5.887268000
H	2.747146000	-0.206877000	3.117595000
H	3.963410000	-0.341553000	-4.424100000
H	4.545899000	-0.372683000	1.146087000
H	4.809382000	-2.162176000	-2.789688000
H	5.176708000	1.429541000	-0.473745000
H	7.273533000	-2.265652000	-3.033512000

H	7.642063000	1.336219000	-0.704008000
H	8.705063000	-0.514707000	-1.989089000
H	-1.163244000	1.808604000	3.777315000
H	-1.166342000	1.806109000	6.249880000
H	-1.702909000	0.133019000	-5.887268000
H	-2.747146000	0.206877000	3.117595000
H	-3.963410000	0.341553000	-4.424100000
H	-4.545899000	0.372683000	1.146087000
H	-4.809382000	2.162176000	-2.789688000
H	-5.176708000	-1.429541000	-0.473745000
H	-7.273533000	2.265652000	-3.033512000
H	-7.642063000	-1.336219000	-0.704008000
H	-8.705063000	0.514707000	-1.989089000
N	0.000000000	0.000000000	7.826082000
N	0.368310000	3.040304000	-1.179557000
N	1.217396000	-0.152732000	-2.620238000
N	1.409089000	-0.166262000	0.089487000
N	-0.368310000	-3.040304000	-1.179557000
N	-1.217396000	0.152732000	-2.620238000
N	-1.409089000	0.166262000	0.089487000
O	0.621140000	-0.902661000	8.429147000
O	-0.621140000	0.902661000	8.429147000

C2: {Co[(5,15-*p*NO$_2$P)(10-P)C](CN)$_2$}$^{2-}$

Co	0.000000000	0.000000000	0.432977000
C	0.000000000	0.000000000	-2.859927000
C	0.000000000	0.000000000	-4.347005000
C	0.000000000	0.000000000	-7.163814000
C	0.096583000	1.890835000	0.422969000
C	0.635489000	1.024596000	-5.065380000
C	0.639691000	1.023778000	-6.460110000
C	0.707027000	-0.037603000	3.114109000
C	1.245069000	-0.053044000	-2.202958000
C	1.780462000	-0.142905000	4.046228000
C	2.539708000	-0.126138000	-2.828273000
C	2.582772000	-0.147218000	1.906266000
C	2.762823000	-0.201899000	-0.569464000
C	2.947908000	-0.221551000	3.300020000
C	3.354509000	-0.240995000	0.722967000
C	3.470472000	-0.201220000	-1.826489000
C	4.807148000	-0.390815000	0.840900000
C	5.503370000	-1.386661000	0.119934000
C	5.553307000	0.447156000	1.698723000
C	6.874071000	-1.533512000	0.233279000
C	6.924506000	0.312775000	1.823917000
C	7.583046000	-0.678253000	1.085873000
C	-0.096583000	-1.890835000	0.422969000
C	-0.635489000	-1.024596000	-5.065380000
C	-0.639691000	-1.023778000	-6.460110000
C	-0.707027000	0.037603000	3.114109000
C	-1.245069000	0.053044000	-2.202958000
C	-1.780462000	0.142905000	4.046228000
C	-2.539708000	0.126138000	-2.828273000
C	-2.582772000	0.147218000	1.906266000
C	-2.762823000	0.201899000	-0.569464000
C	-2.947908000	0.221551000	3.300020000
C	-3.354509000	0.240995000	0.722967000
C	-3.470472000	0.201220000	-1.826489000
C	-4.807148000	0.390815000	0.840900000
C	-5.503370000	1.386661000	0.119934000
C	-5.553307000	-0.447156000	1.698723000
C	-6.874071000	1.533512000	0.233279000
C	-6.924506000	-0.312775000	1.823917000
C	-7.583046000	0.678253000	1.085873000
H	0.000000000	0.000000000	-8.251423000
H	1.127009000	1.824426000	-4.519299000
H	1.136703000	1.828014000	-6.998385000
H	1.688910000	-0.167506000	5.124802000
H	2.717569000	-0.115421000	-3.892984000
H	3.952458000	-0.330511000	3.681713000
H	4.543118000	-0.240307000	-1.938572000
H	4.945285000	-2.065447000	-0.514806000
H	5.041664000	1.231002000	2.245573000

H	7.400411000	-2.308585000	-0.310507000
H	7.495234000	0.973419000	2.465272000
H	-1.127009000	-1.824426000	-4.519299000
H	-1.136703000	-1.828014000	-6.998385000
H	-1.688910000	0.167506000	5.124802000
H	-2.717569000	0.115421000	-3.892984000
H	-3.952458000	0.330511000	3.681713000
H	-4.543118000	0.240307000	-1.938572000
H	-4.945285000	2.065447000	-0.514806000
H	-5.041664000	-1.231002000	2.245573000
H	-7.400411000	2.308585000	-0.310507000
H	-7.495234000	-0.973419000	2.465272000
N	0.157641000	3.060489000	0.426734000
N	1.230337000	-0.044939000	1.854033000
N	1.419860000	-0.092258000	-0.843151000
N	9.013341000	-0.818074000	1.203566000
N	-0.157641000	-3.060489000	0.426734000
N	-1.230337000	0.044939000	1.854033000
N	-1.419860000	0.092258000	-0.843151000
N	-9.013341000	0.818074000	1.203566000
O	9.590373000	-1.667743000	0.498139000
O	9.621332000	-0.082630000	2.004829000
O	-9.590373000	1.667743000	0.498139000
O	-9.621332000	0.082630000	2.004829000

C3: $\{Co[T\textit{p}NO_2PC](CN)_2\}^{2-}$

Co	0.000000000	0.000000000	0.946102000
C	0.000000000	0.000000000	-2.353906000
C	0.000000000	0.000000000	-3.830833000
C	0.000000000	0.000000000	-6.636154000
C	0.083600000	1.879734000	0.934451000
C	0.707661000	-0.035178000	3.628041000
C	0.732827000	0.961714000	-4.559042000
C	0.737087000	0.970004000	-5.946381000
C	1.250591000	-0.036565000	-1.690352000
C	1.780471000	-0.141115000	4.562364000
C	2.548578000	-0.126684000	-2.311300000
C	2.583105000	-0.150966000	2.421586000
C	2.764256000	-0.202342000	-0.051496000
C	2.948806000	-0.225258000	3.815614000
C	3.355255000	-0.251349000	1.239488000
C	3.475794000	-0.215419000	-1.305073000
C	4.809619000	-0.431325000	1.368137000
C	5.486671000	-1.460642000	0.676210000
C	5.569760000	0.399919000	2.220595000
C	6.851881000	-1.653722000	0.821935000
C	6.936417000	0.222569000	2.373686000
C	7.574283000	-0.807468000	1.672104000
C	-0.083600000	-1.879734000	0.934451000
C	-0.707661000	0.035178000	3.628041000
C	-0.732827000	-0.961714000	-4.559042000
C	-0.737087000	-0.970004000	-5.946381000
C	-1.250591000	0.036565000	-1.690352000
C	-1.780471000	0.141115000	4.562364000
C	-2.548578000	0.126684000	-2.311300000
C	-2.583105000	0.150966000	2.421586000
C	-2.764256000	0.202342000	-0.051496000
C	-2.948806000	0.225258000	3.815614000
C	-3.355255000	0.251349000	1.239488000
C	-3.475794000	0.215419000	-1.305073000
C	-4.809619000	0.431325000	1.368137000
C	-5.486671000	1.460642000	0.676210000
C	-5.569760000	-0.399919000	2.220595000
C	-6.851881000	1.653722000	0.821935000
C	-6.936417000	-0.222569000	2.373686000
C	-7.574283000	0.807468000	1.672104000
H	1.287253000	1.722068000	-6.498949000
H	1.288021000	1.721372000	-4.019700000
H	1.687788000	-0.165205000	5.640836000
H	2.737151000	-0.130627000	-3.373755000
H	3.953388000	-0.336940000	4.196382000
H	4.548046000	-0.274191000	-1.412937000
H	4.919237000	-2.131935000	0.041399000
H	5.075109000	1.209395000	2.745740000
H	7.360441000	-2.457379000	0.302956000

H	7.516338000	0.875941000	3.014522000
H	-1.287253000	-1.722068000	-6.498949000
H	-1.288021000	-1.721372000	-4.019700000
H	-1.687788000	0.165205000	5.640836000
H	-2.737151000	0.130627000	-3.373755000
H	-3.953388000	0.336940000	4.196382000
H	-4.548046000	0.274191000	-1.412937000
H	-4.919237000	2.131935000	0.041399000
H	-5.075109000	-1.209395000	2.745740000
H	-7.360441000	2.457379000	0.302956000
H	-7.516338000	-0.875941000	3.014522000
N	0.000000000	0.000000000	-8.083136000
N	0.139005000	3.050069000	0.936042000
N	1.230292000	-0.041210000	2.368425000
N	1.419836000	-0.078432000	-0.332272000
N	8.997843000	-1.001946000	1.832141000
N	-0.139005000	-3.050069000	0.936042000
N	-1.230292000	0.041210000	2.368425000
N	-1.419836000	0.078432000	-0.332272000
N	-8.997843000	1.001946000	1.832141000
O	0.679082000	0.853706000	-8.676094000
O	9.550856000	-1.904647000	1.181981000
O	9.613723000	-0.259738000	2.615146000
O	-0.679082000	-0.853706000	-8.676094000
O	-9.550856000	1.904647000	1.181981000
O	-9.613723000	0.259738000	2.615146000
